# A systematic review of the burden of hypertension, access to services and patient views of hypertension in humanitarian crisis settings

**DOI:** 10.1136/bmjgh-2020-002440

**Published:** 2020-11-09

**Authors:** James Keasley, Oyinlola Oyebode, Saran Shantikumar, William Proto, Majel McGranahan, Amar Sabouni, Farah Kidy

**Affiliations:** 1 Warwick Medical School, University of Warwick, Coventry, UK; 2 Department of Health Sciences, University of York, York, North Yorkshire, UK

**Keywords:** systematic review, hypertension, public health, health policy, epidemiology

## Abstract

**Introduction:**

Globally, a record number of people are affected by humanitarian crises caused by conflict and natural disasters. Many such populations live in settings where epidemiological transition is underway. Following the United Nations high level meeting on non-communicable diseases, the global commitment to Universal Health Coverage and needs expressed by humanitarian agencies, there is increasing effort to develop guidelines for the management of hypertension in humanitarian settings. The objective was to investigate the prevalence and incidence of hypertension in populations directly affected by humanitarian crises; the cascade of care in these populations and patient knowledge of and attitude to hypertension.

**Methods:**

A literature search was carried out in five databases. Grey literature was searched. The population of interest was adult, non-pregnant, civilians living in any country who were directly exposed to a crisis since 1999. Eligibility assessment, data extraction and quality appraisal were carried out in duplicate.

**Results:**

Sixty-one studies were included in the narrative synthesis. They reported on a range of crises including the wars in Syria and Iraq, the Great East Japan Earthquake, Hurricane Katrina and Palestinian refugees. There were few studies from Africa or Asia (excluding Japan). The studies predominantly assessed prevalence of hypertension. This varied with geography and age of the population. Access to care, patient understanding and patient views on hypertension were poorly examined. Most of the studies had a high risk of bias due to methods used in the diagnosis of hypertension and in the selection of study populations.

**Conclusion:**

Hypertension is seen in a range of humanitarian settings and the burden can be considerable. Further studies are needed to accurately estimate prevalence of hypertension in crisis-affected populations throughout the world. An appreciation of patient knowledge and understanding of hypertension as well as the cascade of care would be invaluable in informing service provision.

**Key questions**

**What is already known?**

- Globally, non-communicable diseases are a leading cause of mortality and according to the WHO, hypertension is estimated to cause 12.8% of all deaths.
- As of 2018, more than 70 million people were displaced as a result of humanitarian crises.
- It is likely that people with hypertension are underserved in humanitarian settings.

**What are the new findings?**

- Reported prevalence of hypertension in crisis-affected populations varies considerably across different populations with higher prevalence in those living in high-income countries.
- There are very little data from Africa and Asia (except Japan) despite protracted refugee situations on these continents.
- We have collated published estimates of hypertension prevalence by crisis, as well as the limited data on hypertension control, access to care and treatment, and patient understanding of hypertension.

**What do the new findings imply?**

- Given the substantial prevalence of hypertension in many of the studies, assessment of hypertension and appropriate resourcing to treat it should be a priority for agencies providing emergency and longer-term care for patients after or during humanitarian crises in order to prevent significant mortality and morbidity.
- There is a need to capture the patient experience in order to better inform service development.
- Further research is needed, especially in Africa and Asia where there are additional protracted refugee situations.

## Introduction

Non-communicable diseases (NCDs) are the leading cause of mortality globally, and nearly three-quarters of deaths due to NCDs occur in low/middle-income countries (LMICs).^1^

The Humanitarian Coalition defines a humanitarian crisis as ‘an event or series of events that represents a critical threat to the health, safety, security or well-being of a community or other large group of people’.^2^ Globally, the number of people affected by persecution, conflict, violence, human rights violations or natural disasters remains at record high levels; there were 70.8 million people displaced at the end of 2018. Increasing numbers of refugees are living in situations of protracted displacement (average 20 years) and in urban settings. Nine out of the ten countries hosting most of the world’s refugee populations are themselves developing nations and as such face financial and infrastructure challenges to meeting healthcare and other needs.^3^

Large numbers of those experiencing forced displacement (refugees or internally displaced persons) originate from Syria, Colombia, South Sudan, Democratic Republic of Congo, Myanmar, Yemen, Ethiopia and Somalia.^3^ In the former two countries, the epidemiological transition was apparent even before the displacement occurred, with cardiovascular diseases being the main cause of death in 1990. Even now, ischaemic heart disease and stroke combined contribute a similar proportion of deaths as conflict and terrorism in Syria, 33.83% and 36.13%, respectively.^4^

These factors have led to increasing calls for consideration of NCD care in humanitarian crises.^5–7^ The United Nations (UN) high-level meeting on the prevention and control of NCDs in 2011 highlighted the importance of prevention^8^ and workshops with non-governmental organisations (NGOs) and civil society groups highlighted the need for further information about prevention activities in humanitarian settings in particular.^9^ UN member states have agreed to achieve Universal Health Coverage (UHC) (which includes healthcare in humanitarian crises) by 2030, and prevention and treatment of raised blood pressure (BP) is one of the key recommended indicators.^10^

We can expect to find both new and poorly controlled hypertension in humanitarian crises. Armed conflicts in particular, are associated with increased short-term and long-term cardiac morbidity and mortality and increases in BP.^11 12^ Furthermore, the concept of ‘disaster hypertension’ was coined to describe the, often transient, increase in BP and associated cardiovascular mortality seen after natural disasters.^13^ For NCD management in general, disrupted supply of medication and the challenge of keeping already pressurised health systems functioning, are likely to result in poor disease control for existing patients.^5^ For hypertension in particular, there is ongoing research examining the link between different types of stress and increases in BP. Chronic rather than acute stress is associated with the development of long-term hypertension^14 15^ and there is some suggestion that higher levels of stress are associated with increased risk of developing the disease.^16^ Following exposure to conflict, research in military populations shows that post-traumatic stress disorder and injury severity are independent risk factors for the development of hypertension.^17^

Existing reviews have examined the burden of diabetes,^18^ substance misuse,^19 20^ smoking,^21^ alcohol,^22 23^ cardiovascular disease^11^ and NCDs^24 25^ in specific humanitarian settings. However, we do not have a broad understanding of hypertension in populations affected by crises. This is needed for service development.

The objective of this review was to investigate the prevalence and incidence of hypertension in populations directly affected by humanitarian crises; the cascade of care in these populations and patient knowledge of and attitude to hypertension.

## Methods

We carried out a systematic review in accordance with Preferred Reporting Items for Systematic Reviews and Meta-Analyses (PRISMA) guidelines.^26^ Five databases were searched: Medline, Embase, PsycINFO, Cumulative Index of Nursing and Allied Health Literature and Web of Science. The search terms were adapted, following consultation with a librarian, from a similar review by Kehlenbrink *et al*^18^ examining the available evidence for the burden of diabetes in humanitarian crises. The search strategy is outlined in online supplemental appendix 1. Searches were conducted on 19th of August 2019. Given the importance of NGOs and civil service organisations in this area, the following grey literature databases were searched on 28th of August 2019: Google, ReliefWeb, UN High Commissioner for Refugees, WHO Institutional Repository for Information Sharing, UNICEF, Médecins Sans Frontières, International Rescue Committee, International Committee of the Red Cross, Centre for Disease Control and Prevention and Active Learning Network for Accountability and Performance. A list of the search terms used to identify relevant grey literature can be seen in online supplemental appendix 2.

10.1136/bmjgh-2020-002440.supp1 Supplementary data

10.1136/bmjgh-2020-002440.supp2 Supplementary data

Studies were assessed for the following criteria:

### Population

The population of interest was non-pregnant, civilian adults (defined as 18 years or older) who had been directly exposed to a humanitarian crisis. This included refugees and internally displaced people.

### Exposure

For the purposes of this review, the causes of humanitarian crises of interest were author defined armed conflict, complex emergencies and natural disasters (including earthquakes, floods, landslides, tidal waves, tsunamis, cyclones, droughts and famine). Only those studies reporting exposure which was ongoing in or began after 1 January 1999 were included.

### Comparator

The presence or absence of a comparator was not used to determine study inclusion.

### Outcome

The primary outcomes of interest were:

1. Prevalence and incidence of hypertension in populations directly affected by conflict, complex emergencies or natural disasters.
2. Proportion diagnosed with hypertension who are aware of the diagnosis, are receiving treatment, support or advice at time of study, and have achieved adequate levels of control.
3. Proportion with known hypertension who sought treatment but did not receive it.
4. Patient knowledge and attitude of hypertension as a problem.

Secondary outcomes were:

1. Understanding of whether or not hypertension management is included as part of a wider programme of prevention or health promotion.
2. Barriers and facilitators to accessing treatment.

For inclusion purposes, there were no restrictions placed on the mode of diagnosis of hypertension, duration of illness or treatment received.

#### Eligibility criteria

We included all study types, in any language, set in high-income, middle-income or low-income countries. Studies of populations directly affected by crises including general populations or populations selected from service users (without restriction by disease type) or selected for hypertension or cardiovascular disease interventional studies were included. Studies with mixed populations were only included if the population of interest could be clearly differentiated. Qualitative studies were included if patients with hypertension from the population of interest were directly interviewed.

Theses, conference proceedings, letters, clinical guidelines, study protocols, reports with no description of methods, opinion pieces or any study published before 1 January 1999 were excluded. Studies relating to economic migrants or migrants not affected by conflict or natural disasters were excluded. The individual study author’s definitions of type of migrant and exposure were applied. Studies including children (aged under 18 years) or examining hypertension in pregnancy where non-pregnant, adult data could not be extracted were excluded. Studies including only military or service personnel were also excluded. Isolated incidents of terrorism out with the context of armed conflict were not considered eligible.

**Figure 1:**
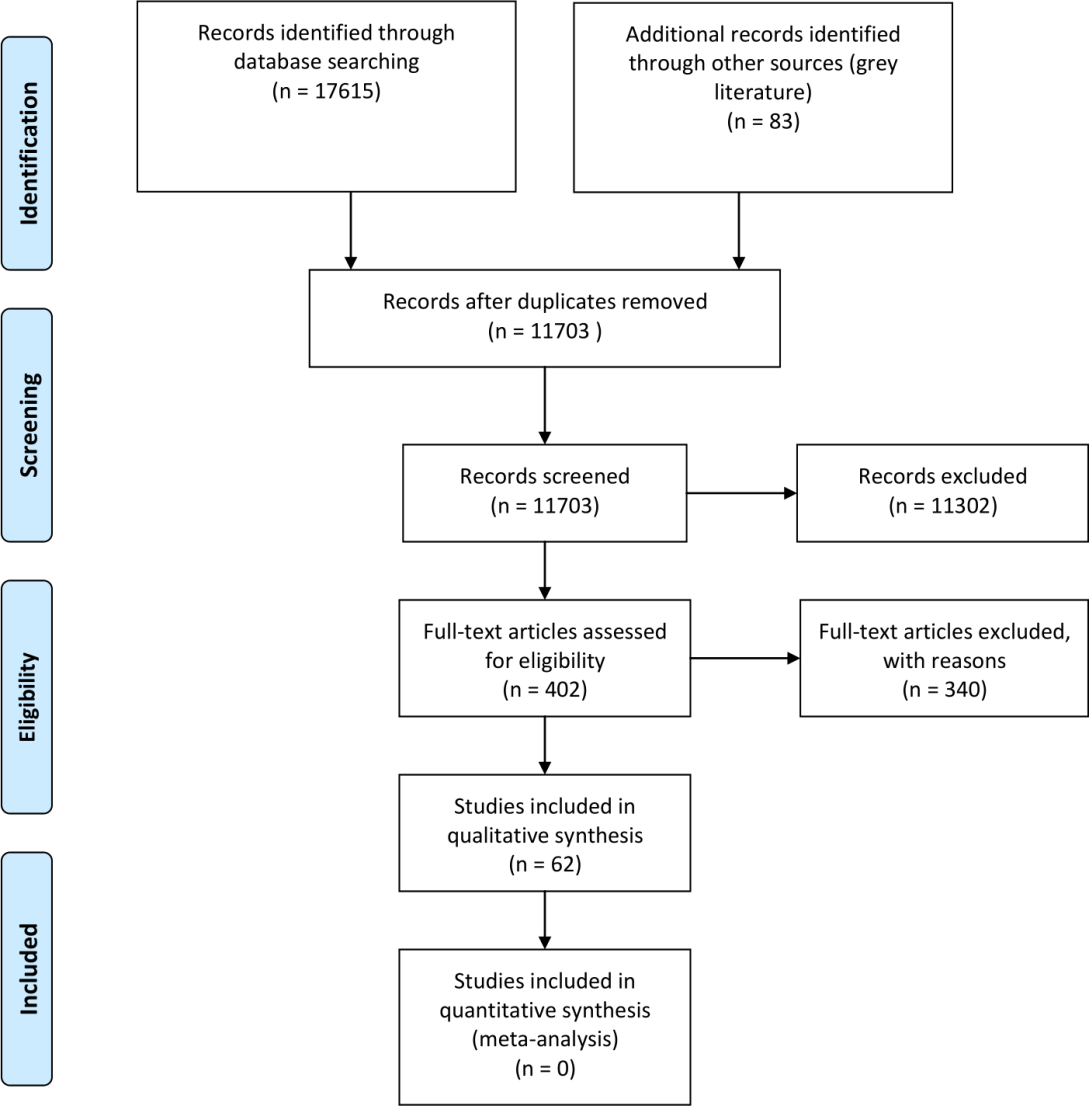
PRISMA flow diagram. From: Moher D, Liberati A, Tetzlaff J, Altman DG, The PRISMA Group (2009). Preferred Reporting Items for Systematic Reviews and Meta-Analyses: The PRISMA Statement. PLoS Med 6(7): e1000097. doi:10.1371/journal.pmed1000097. For more information, visit www.prisma-statement.org.

The authors used Rayyan to manage the identified references.^27^ Two reviewers screened the titles and abstracts of identified studies against the inclusion/exclusion criteria. At this stage, if either reviewer included a study, it was taken through to the full-text screening. Full texts of 318 published studies and 83 grey literature articles were screened for eligibility. Any conflicts were discussed and consensus was arrived at. Any studies excluded at the full-text screening stages were documented in a table with accompanying reasoning (online supplemental appendices 3 and 4). A third reviewer did not need to be consulted to resolve any disagreements. Two studies could not be accessed and so were excluded. The screening process is outlined in the PRISMA flow diagram in figure 1.

10.1136/bmjgh-2020-002440.supp3 Supplementary data

10.1136/bmjgh-2020-002440.supp4 Supplementary data

#### Definitions

Any definition of hypertension was considered, as described in individual studies. However, on quality assessment, the authors considered acceptable definitions of hypertension to be the WHO definition (ie, diagnosis if, when it is measured on two different days, the systolic BP readings on both days was ≥140 mm Hg and/or the diastolic BP readings on both days was ≥90 mm Hg.); or national or international clinical guidelines specified in a study; or doctor diagnosed hypertension; or the use of anti-hypertensive medication in those with a history of hypertension.

#### Data extraction and risk of bias assessment

Data extraction and risk of bias assessment were carried out by one author (JK or OO) with a 10% cross check by a second author (JK or FK). A data extraction tool was developed and piloted. For all study types, a description of the population, the location of the study, the type of study and a description of the crisis were extracted. For quantitative studies, the definition of hypertension and the number and proportion of those with the disease (with a measure of spread) for the whole population and for population subgroups were extracted. For qualitative studies, concepts and themes around the patients’ understanding of hypertension (primary outcome) and barriers and facilitators (secondary outcomes) were extracted.

**Figure 2:**
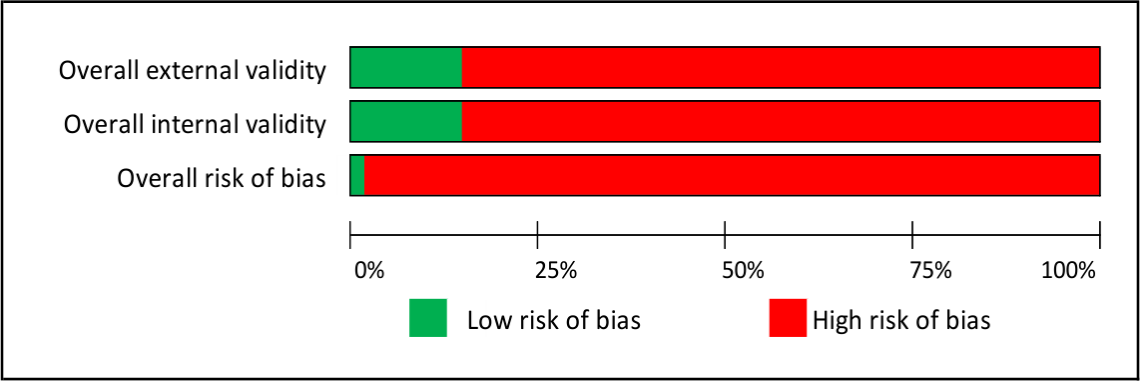
Risk of bias. External validity questions: Was the sampling frame a true or close representation of the target population?, Was some form of random selection used to select the sample, or was a census undertaken?, Was the likelihood of non-response bias minimal? Internal validity questions: Were data collected directly from the subjects (as opposed to a proxy)?, Was an acceptable case definition used?, Was the study instrument that measured the parameter of interest shown to have reliability and validity?, Was the same mode of data collection used for all subjects?, Was the length of the shortest prevalence period for the parameter of interest appropriate?, Were the numerator and denominator for the parameter of interest appropriate?

A tool developed by Hoy *et al*^28^ was used for assessing risk of bias in prevalence studies. This tool assesses external and internal validity. The external validity domain comprises questions about the sampling frame, sample selection and non-response bias. The internal validity domain comprises questions about the case definition, the measurement tool selected and application of that tool (see figure 2 for more details.) After piloting the tool, the authors adapted it slightly in order to give the studies a rating (of either high or low risk of bias) for both internal and external validity, as well as an overall score. This added further detail for assessing the quality of the included studies. For qualitative papers, the CASP toolkit was used.^29^

Due to the heterogeneity of the included studies, the results were synthesised in a narrative description, exploring disease burden, treatment provision and treatment uptake. None of the qualitative papers we have included reported on our primary outcomes. Had there been more data available, we planned an interpretative review using a meta-ethnographic approach.^30^

#### Patient and public involvement

This research was done without patient or public involvement.

## Results

### Study characteristics

**Figure 3:**
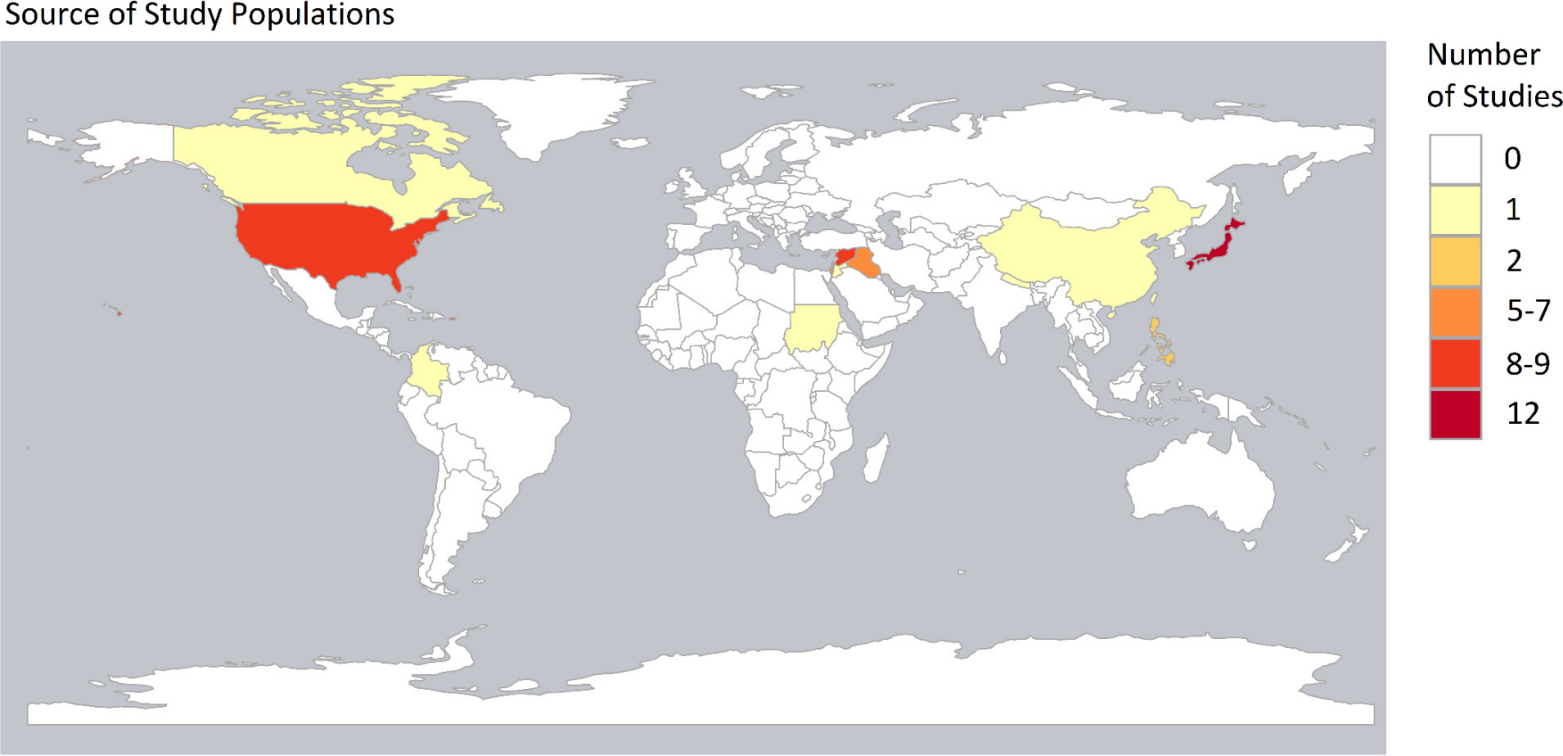
Map showing the number of papers by country of origin of study population. Map created using R. Granada is missing from the map and featured in one paper.

Sixty-two papers reporting 61 studies were included in the analysis. Table 1 outlines the key features of the included studies. The papers are organised by alphabetical order of the crisis. Twenty-five relate to conflicts, 6 relate to long-standing refugee situations and 31 are non-conflict related. Thirty studies were conducted in high-income countries (HICs) and the other 31 were conducted in LMICs. Of the HIC studies, five were related to populations who had been displaced from LMICs.^31–34^ The map in figure 3 shows the countries from which participants originated. The most frequently examined populations were from Japan, followed by the USA and Syria.

**Table 1:** Study characteristics

First author and year	Description of crisis (name, category and dates, as described in paper)	Population as described in paper	Adults or elderly (as defined by paper)	Displaced	Type of crisis	Location of study
Gómez-Restrepo *et al* 2018^84^	Colombian conflicts, (ongoing)	10764 respondents to the National Mental Health Survey (2016), some of whom exposed to armed conflict	Adult	Unclear	Conflict	LMIC
Furusawa *et al* 2011^85^	Earthquake, Solomon Islands, (2007)	Villagers in Western province	Adult	Mixed	Non-conflict	LMIC
Vanasse *et al* 2016^86^	Flood in the city of Saint-Jean-sur-Richelieu, (2011)	All adult individuals covered by the Quebec universal public health insurance plan resident in postcodes affected by the flood (271 postcodes, 119 of which had more than half of their surface area flooded)	Adult	Unclear	Non-conflict	HIC
Ebner *et al* 2016^38^	Great East Japan Earthquake, (2011)	Adults over 40 living in Kawauchi village, 20 km west from Fukushima	Adult	Yes	Non-conflict	HIC
Hayashi *et al* 2017^87^	Great East Japan Earthquake, (2011)	People aged 40–74 years living in 1 of 13 municipalities in Fukushima in the evacuation zone	Adult	Mixed	Non-conflict	HIC
Hoshide *et al* 2019^88^	Great East Japan Earthquake, (2011)	388 evacuees living in evacuation shelters after the earthquake	Adult	Yes	Non-conflict	HIC
Nagai *et al* 2018^37^	Great East Japan Earthquake, (2011)	Patients aged 40–74 years living near Fukushima Daiichi Nuclear Power Plant	Adult	Mixed	Non-conflict	HIC
Nomura *et al* 2016^89^	Great East Japan Earthquake, (2011)	Patients aged 40–74 years living in Minamisoma and Soma	Adult	Mixed	Non-conflict	HIC
Ohira *et al* 2016^51^	Great East Japan Earthquake, (2011)	Evacuee and non-evacuee Japanese adults without hypertension aged 40–74 years living near the Fukushima Daiichi Nuclear Power Plant before the earthquake	Adult	Mixed	Non-conflict	HIC
Sakai *et al* 2017^70^	Great East Japan Earthquake, (2011)	Individuals aged 40–90 years living in the vicinity of the Fukushima Daiichi Nuclear Power Plant who had attended annual health checkups since 2008	Adult	Mixed	Non-conflict	HIC
Satoh *et al* 2016^39^	Great East Japan Earthquake, (2011)	Patients aged 40–90 years who were living near the Fukushima Daiichi Nuclear Power Plant	Adult	Mixed	Non-conflict	HIC
Shiba *et al* 2019^90^	Great East Japan Earthquake, (2011)	Patients aged 65 years or older in Iwanuma affected by the tsunami	Elderly	Mixed	Non-conflict	HIC
Suda *et al* 2019^91^	Great East Japan Earthquake, (2011)	Evacuees with disaster medical records from Minamisanriku town after the earthquake	Adult	Mixed	Non-conflict	HIC
Takahashi *et al* 2016^92^	Great East Japan Earthquake, (2011)	6528 disaster survivors in heavily tsunami-damaged municipalities (both relocated and non-relocated)	Adult	Mixed	Non-conflict	HIC
Toda *et al* 2017^93^	Great East Japan Earthquake, (2011)	Adults over 40 years living in Minamisoma city, 10–40 km from the Fukushima Daiichi Nuclear Plant	Adult	Mixed	Non-conflict	HIC
An *et al* 2015^49^	Hurricane Ike, (2008)	Free clinic attendees who had stayed on the island during the rainstorm >18 years, did not have a pre-hurricane history of coronary heart disease or psychiatric diagnoses and could read English	Adult	No	Non-conflict	HIC
Gomez *et al* 2009^94^	Hurricane Ivan, (2004)	Adult members of the community of 'Jubilee', Grenada	Adult	No	Non-conflict	LMIC
Greenough *et al* 2008^95^	Hurricane Katrina, (2005)	Heads of household in Louisiana American Red Cross shelters	Adult	Yes	Non-conflict	HIC
Kessler 2007^96^	Hurricane Katrina, (2005)	Adults living in areas eligible for assistance after Hurricane Katrina	Adult	Mixed	Non-conflict	HIC
Islam *et al* 2008^64^	Hurricane Katrina, (2005)	2194 adults over 65 with hypertension on managed care organisation databases	Elderly	No	Non-conflict	HIC
Krol *et al* 2007^97^	Hurricane Katrina, (2005)	Patients from Biloxi/Gulfport affected by Hurricane Katrina	Adult	Unclear	Non-conflict	HIC
Krousel-Wood *et al* 2008^60^	Hurricane Katrina, (2005)	Community-dwelling patients attending the hypertension section of a multispecialty group practice	Adult	Mixed	Non-conflict	HIC
Rodriguez *et al* 2006^40^	Hurricane Katrina, (2005)	Adult evacuees from Louisiana in Oklahoma	Adult	Yes	Non-conflict	HIC
Vest *et al* 2006^41^	Hurricane Katrina, (2005)	New Orleans adults displaced by Hurricane Katrina, sheltered in Austin, Texas	Adult	Yes	Non-conflict	HIC
Arrieta *et al* 2009^65^	Hurricane Katrina, 2005	30 health and social service providers, 28 chronic disease patients	Adult	Unclear	Non-conflict	HIC
Burton *et al* 2009^35^	Hurricane Katrina, 2005	Non-institutionalised Peoples Health enrollees who lived in four parishes in the New Orleans metropolitan area (age 65+ years)	Elderly	Mixed	Non-conflict	HIC
Burger *et al* 2019^48^	Hurricane Sandy, (2012)	Patients from 7 Federal Quality Health Centres in New Jersey	Adult	No	Non-conflict	HIC
Prueksaritanond *et al* 2007^98^	Indian Ocean earthquake and tsunami, (2004)	87 elderly members of the Ban Nam Khem Community, Thailand	Elderly	No	Non-conflict	LMIC
Doocy *et al* 2013^42^	Iraq War, (2003–2011)	Iraqi populations displaced in Jordan and Syria	Adult	Yes	Conflict	LMIC
Mateen *et al* 2012^99^	Iraq War, (2003–2011)	Iraqi refugees in Jordan	Adult	Yes	Conflict	LMIC
Lafta *et al* 2016^100^	Iraq War and subsequent sectarian violence, (2003–)	Women from internally displaced families living in informal settlements	Adult	Yes	Conflict	LMIC
Anon *et al* 2010 101	Iraq War, (2003–2011)	Iraqi refugees settled in San Diego, California	Adult	Yes	Conflict	HIC
Jen *et al* 2015^31^	Iraq War, (2003–2011)	290 Iraqi adult refugees recently arrived in Michigan	Adult	Yes	Conflict	HIC
Taylor *et al* 2014^32^	Iraq War, (2003–2011)	Iraqi adults settled in the USA for between 8 and 36 months	Adult	Yes	Conflict	HIC
Baxter *et al* 2018^66^	Iraqi Civil War, (2014–17)	15 adult from Mosul presenting to MSF in Kurdistan	Adult	Yes	Conflict	LMIC
Cetorelli *et al* 2017^52^	Iraqi Civil War, (2014–17)	Adults in BRHA camps	Adult	Yes	Conflict	LMIC
Dudova *et al* 2015^102^	Iraqi Civil War, (2014–17)	1119 refugee patients at a primary care clinic for internally displaced people in Ozal city, Erbil	Adult	Yes	Conflict	LMIC
Lipsitz *et al* 2010^36^	Israel–Lebanon War, (2006)	Patients attending five clinics in Jerusalem and surrounding areas, whose home address was in northern Israel	Adult	Yes	Conflict	HIC
Adrega *et al* 2018^103^	Nepal earthquake, (May 2015)	Dislodged inhabitants of Sindhupalchok	Adult	Mixed	Non-conflict	LMIC
Khader *et al* 2014^53^	Palestine conflict, (—)	All Palestinian patients registered in 6 UNRWA primary care clinics in Jordan	Adult	Yes	Long-standing refugee situation	LMIC
Khader *et al* 2012^61^	Palestine conflict, (—)	Palestinian refugees registered with Nuzha primary care centre with hypertension	Adult	Yes	Long-standing refugee situation	LMIC
Mousa *et al* 2010^104^	Palestine conflict, (—)	Palestinian refugees in Jordan, Lebanon, Syrian Arab Republic, West Bank and the Gaza Strip served by UNRWA primary care facilities	Adult	Yes	Long-standing refugee situation	LMIC
Saadeh *et al* 2015^43^	Palestine conflict, (—)	Palestinian refugees (aged 40 years and over) treated by GPs at the UNRWA primary healthcare centres in Jordan	Adult	Yes	Long-standing refugee situation	LMIC
Saleh *et al* 2018 44	Palestine conflict, (—)	Palestinian refugees (aged 40 years and over) living in camps in Lebanon	Adult	Yes	Long-standing refugee situation	LMIC
Abukhdeir *et al* 2013^105^	Palestinian conflict, (—)	Palestinians living in the Gaza Strip and the West Bank	Adult	Mixed	Long-standing refugee situation	LMIC
Renzaho *et al* 2014^33^	Sudanese wars (ongoing throughout time frame)	314 Sudanese refugees settling in Brisbane/Toowoomba, Australia.	Adult	Yes	Conflict	HIC
Mobula *et al* 2016^50^	Super Typhoon Haiyan, Philippines, (2013)	Patients attending clinics conducted by mobile medical teams	Adult	Unclear	Non-conflict	LMIC
Balcilar *et al* 2016^62^	Syrian War, (2011–)	Syrian refugees in Turkey aged 18–69 years	Adult	Yes	Conflict	LMIC
Chahda *et al* 2015^47^	Syrian War, (2011–)	167 Syrian refugees registered with CLMC and 43 Palestinian refugees from Syria registered with the Palestinian Women’s Humanitarian Organisation in Lebanon	Elderly	Yes	Conflict	LMIC
Doocy *et al* 2018^54^	Syrian War, (2011–)	Syrian refugees in Lebanon	Adult	Yes	Conflict	LMIC
Doocy *et al* 2015^67^	Syrian War, (2011–)	Syrian refugees in Jordan	Adult	Yes	Conflict	LMIC
Doocy *et al* 2016^55^	Syrian War, (2011–)	Syrian refugees in Lebanon	Adult	Yes	Conflict	LMIC
Eryurt *et al* 2020^46^	Syrian War, (2011–)	Syrian refugees in Turkey (age 18–69 years)	Adult	Yes	Conflict	LMIC
Maldari *et al* 2019^34^	Syrian War, (2011–)	Syrian refugees seen at Refugee Health Service, South Australia	Adult	Yes	Conflict	HIC
Rehr *et al* 2018^56^	Syrian War, (2011–)	Non-camp Syrian refugees in Irbid northern Jordan	Adult	Yes	Conflict	LMIC
Strong *et al* 2015^106^	Syrian War, (2011–)	210 older refugees from Syria in Lebanon	Elderly	Yes	Conflict	LMIC
Doocy *et al* 2014^107^	Syrian War, (2011–)	Syrian refugees in Jordan	Adult	Yes	Conflict	LMIC
Vernier *et al* 2019^45^	Syrian War, (2011–)	Families living in Ein Issa camp	Adult	Yes	Conflict	LMIC
Doocy *et al* 2016^57^	Syrian War, (2011)	Syrian refugees in Jordan	Adult	Yes	Conflict	LMIC
Kayali *et al* 2019^58^	Syrian War, (2011)	Patients with hypertension in Shatila refugee camp	Adult	Yes	Conflict	LMIC
Lin *et al* 2015^108^	Typhoon Morakot, Taiwan, (2009)	Typhoon-displaced adults from Kaohsiung County. Two groups: 1 moved to shelters, 1 moved within the community	Adult	Yes	Non-conflict	LMIC
Sun *et al* 2013^59^	Wenchuan earthquake, China (2008)	3230 adults over 20 who had stayed in temporary shelters for more than 1 year	Adult	Yes	Non-conflict	LMIC

BRHA, Board of Relief and Humanitarian Affairs; CHD, coronary heart disease; CLMC, Caritas Lebanon Migrant Centre; GP, general practitioner; HIC, high-income country; LMIC, low/middle-income country; MSF, Médecins Sans Frontières; UNRWA, United Nations Relief and Works Agency for Palestinian Refugees in the Middle East.

Six papers related to elderly populations and the remaining papers were related to general adult populations over the age of 18 years. Thirty-five studies considered displaced populations, while 5 considered non-displaced populations and 16 considered mixed populations of both displaced and non-displaced participants. Displacement status was unclear for five studies.

Several crises were the subject of multiple studies. The Great East Japan Earthquake was the subject of 12 studies, Hurricane Katrina was the subject of 9 studies, the Iraq wars since 2003 were the subject of 9 studies, the Palestinian conflict was the subject of 6 studies and the Syrian War was the subject of 12 studies (13 papers).

### Prevalence and incidence of hypertension

Fifty-five studies reported information on hypertension prevalence. Of these, only 33 have prevalence rates related to the whole population sample. The remaining papers measure prevalence in subgroups, either by sex, age, ethnicity, evacuation status, location or housing damage. Among the whole population samples, the prevalence rates range from 3%^35 36^ to 83.14%.^35^ The wide range reflects the heterogeneity of the studies, especially the different mean ages of the populations. We present the prevalence data in tables 2 and 3 by country income levels of HICs or LMICs.

**Table 2:** Prevalence of hypertension reported in eligible studies conducted in high-income countries

First author and year	Measurement used	Subgroup (if no whole population prevalence available)	Number with disease	Value of denominator	Proportion with disease as reported (%)	Measure of spread as reported (95% CI)
**Japan**						
Ebner *et al* 2016^38^	BP >130/85 or on antihypertensive medications	Pre-earthquake	6107	9296	65.7	62.7 to 68.5
		2012	1731	2801	61.8	58.2 to 65.2
		2013	1780	2794	63.7	59.9 to 67.4
Hayashi *et al* 2017^87^	BP >140/90 or on medication	Baseline BP		14 492	54	
Hoshide *et al* 2019^88^	Mean of 3×BP >140/90	Disaster HTN	158	272		
		Pre-earthquake HTN	126	272		
		Disaster HTN among patients without prevalent HTN	66	146	45.2	
		Disaster HTN among patients with HTN normally	92	126	73	
Nagai *et al* 2018^37^	BP >140/90 or on antihypertensive medications	2010 male		9912	44.4	42.7 to 46.0
		2011 male		7249	47.2	45.2 to 49.2
		2012 male		9499	48.8	47.1 to 50.6
		2013 male		9485	47.4	45.7 to 49.2
		2014 male		9619	45.5	43.8 to 47.3
		2010 female		12 178	37.5	36.3 to 38.7
		2011 female		8828	38.6	37.3 to 40.0
		2012 female		11 615	39	37.8 to 40.3
		2013 female		11 851	37.8	36.6 to 39.1
		2014 female		12 341	35.6	34.4 to 36.8
Nomura *et al* 2016^89^	BP >140/90 or on antihypertensive medications	Baseline (2008–2010) evacuee	437	960	45.5	
		Baseline (2008–2010) non-evacuee	2463	5446	45.3	
		2011 evacuee	112	216	51.9	
		2011 non-evacuee	1506	2870	52.6	
		2012 evacuee	333	627	53.1	
		2012 non-evacuee	2020	3886	52.1	
		2013 evacuee	366	657	55.7	
		2013 non-evacuee	1888	3680	51.3	
		2014 evacuee	297	617	48.1	
		2014 non-evacuee	1758	3591	49.2	
Ohira *et al* 2016^51^	On medication or BP >140/90	Men	1242	4515	27.5	
		Women	1362	6522	20.9	
Sakai *et al* 2017^70^	Not stated	Evacuee		5364	54.1	
		Non-evacuee		2349	56.3	
Satoh *et al* 2016^39^	Not stated	Low risk, evacuee		7253	56.3	
		Low risk, non-evacuee		13 730	56.1	
		Moderate risk, evacuee		1777	71	
		Moderate risk, non-evacuee		3319	69.5	
		High risk, evacuee		290	86.6	
		High risk, non-evacuee		492	80.3	
		Very high risk, evacuee		87	93.1	
		Very high risk, non-evacuee		140	92.1	
Shiba *et al* 2019^90^	HTN medication		414	1195	34.6	
Suda *et al* 2019^91^	ICD-10		2678	10 462	25.6	
Takahashi *et al* 2016^92^	Self-reported			3160	32	
				3368	29	
Toda *et al* 2017^93^	Antihypertensive medication	2009 men	74	224	33	
		2009 women	85	339	25.1	
		2010 men	80	224	35.7	
		2010 women	96	339	28.3	
		2011 men	90	224	40.2	
		2011 women	109	339	32.2	
		2012 men	99	224	44.2	
		2012 women	124	339	36.6	
**Hurricane Katrina**						
Greenough *et al* 2008^95^	Self-reported		170	499	34.8	30.4 to 39.2
Kessler 2007^96^	Self-reported			1043	31.2	SE±2.5
Krol *et al* 2007^97^	Record on medical chart	Age 22–65	99	677	26.1	
		Age >65	29	72	59.2	
Rodriguez *et al* 2006^40^	Self-reported		62	241	25.7	
Vest *et al* 2006^41^	Self-reported		71	183	39.5	32.5 to 46.5
Burton *et al* 2009^35^	ICD-9-CM			20 612	83.14	
**Other HIC**						
Vanasse *et al* 2016 (Canada)^86^	History of hypertension was defined as a hospitalisation with a main or a secondary diagnosis of hypertension (ICD-9 codes: 401–405 and ICD-10-CA codes: I10–I13, I15) or at least two ambulatory visits for hypertension in a 2-year span between 1996 and the beginning of the study period	2328	Whole sample	10 081	23.1	
An *et al* 2015 (USA)^49^	Self-reported/on medication/average of 3 consecutive measures, cut-off >140/90		14	19	73.7	
Burger *et al* 2019 (USA)^48^	Self-reported		27	489	5.5	

BP, blood pressure; HIC, high-income country; HTN, hypertension; ICD, International Classification of Diseases.

**Table 3:** Prevalence of hypertension reported in eligible studies conducted in LMICs

First author and year	Measurement used	Subgroup (if no whole population prevalence available)	Number with disease	Value of denominator	Proportion with disease as reported (%)	Measure of spread as reported (95% CI)
**Iraq**						
Doocy *et al* 2013^42^	Taking medications or medical visits	Jordan		3414	19.6	18.3 to 21.0
		Syria		2342	19.6	18 to 21.3
Mateen *et al* 2012^99^	ICD-10 diagnosis	18–59 male	390	2252	17.3	
		18–59 female	420	2399	17.5	
		60–79	784	1230	63.7	
		>80	76	100	76	
Lafta *et al* 2016^100^	Diagnosis by a health worker		240	1216	19.7	
Anon *et al* 2010^101^	BP >140/90 on two measurements		83	129	64.3	
Jen *et al* 2015^31^	Doctor diagnosed		28	290	9.6	
			38	290	13.1	
Taylor *et al* 2014^32^	Self-reported		95	366	26	
Cetorelli *et al* 2017^52^	Health professional diagnosis	30–44		1193	4.4	3.2 to 5.9
		45–59		550	23.9	19.8 to 28.5
		60+		309	32.1	26.3 to 38.4
Dudova *et al* 2015^102^	On patient documents	35–44	10	122	8.2	
		45–54	46	204	22.6	
		55–64	79	231	34.2	
		65+	109	226	48.2	
**Palestine**						
Saadeh *et al* 2015^43^	Clinical diagnosis	2008	42 495	307 935	13.8	
		2009	46 317	312 951	14.8	
		2010	49 361	324 743	15.2	
		2011	52 718	323 423	16.3	
		2012	53 278	367 434	14.5	
Mousa *et al* 2010^104^	One-off BP >140/90		1453	7762	18.7	
Abukhdeir *et al* 2013^105^	Self-reported	Age 20–39	77		0.8	
		Age 40–64	633		14.1	
		Age 65+	393		33.1	
Saleh *et al* 2018^44^	One-off BP >140/90				37.2	
**Syria**						
Balcilar *et al* 2016^62^	>140/90 or on medication		1463	5713	25.6	24.4 to 26.7
Chahda *et al* 2015^47^	Self-reported	Elderly Syrian refugees	89	167	53	46 to 61
		Elderly Palestinian refugees from Syria	37	43	86	72 to 94
Doocy *et al* 2015^67^	Self-reported	18–39	60	3019	2	1.4 to 2.6
		40–59	220	1040	21.1	18.6 to 23.7
		60+	195	374	52.1	46.5 to 57.7
		Adult	475	4433	10.7	9.8 to 11.7
Doocy *et al* 2016^55^	Healthcare professional diagnosed			3886	7.4	6.6 to 8.3
Eryurt *et al* 2020^46^	Medication for hypertension or raised BP (average of 2 readings)		1687	5322	32	
Maldari *et al* 2019^34^	Patient/clinician reported		25	186	13.4	
Rehr *et al* 2018^56^	Self-reported		1126	8029	14	13.2 to 14.8
Doocy *et al* 2014107	Self-reported		500	5154	9.7	8.8 to 10.6
Vernier *et al* 2019^45^	Self-reported		28	582	4.8	3.3 to 6.9
**Other LMIC**						
Sun *et al* 2013 (China)^59^	BP >140/90 or diagnosis or taking medications		778	3230	24.08	
Gomez-Restrepo *et al* 2018 (Colombia)^84^	Doctor diagnosed	Exposed to conflict	101	493	20.4	15.7 to 26.1
	Doctor diagnosed	Exposed to conflict and another traumatic event	65	346	18.7	12.2 to 27.6
Adrega *et al* 2018 (Nepal)^103^	Self-reported/on medication/one-off BP >140/90		62	167	22.4	
Mobula *et al* 2016 (Philippines)^50^	BP >=140/90		1709	3633	47	
Renzaho *et al* 2014 (Sudan)^33^	Healthcare diagnosed		39	314	12.4	
Lin *et al* 2015 (Philippines)^108^	Self-reported		127	228	46.2	
Prueksaritanond *et al* 2007 (Taiwan)^98^	Not reported		38	87	43.7	
Furusawa *et al* 2011 (Solomon Islands)^85^	One-off BP >140/90	Titiana	79	622	12.7	
		Tapurai	68	919	7.4	
		Mondo	59	129	45.8	
Gomez *et al* 2009 (Grenada)^94^	Not reported				30	

BP, blood pressure; ICD, International Classification of Diseases; LMIC, low/middle-income country.

Of the groups of studies, the Great East Japan Earthquake studies (table 2) are the most homogenous because 8 of the 12 studies gathered data from the same source of routine health data targeting adults between the ages of 40 and 74 years. The prevalence range in these studies was 35.6% in female participants in 2014^37^ to 65.7% in both men and women before the earthquake.^38^ Several of the studies examined the changing prevalence of hypertension from the years before the earthquake to the years following the earthquake. Among these studies, some of the data may have been repeated because the authors used samples from overlapping communities. However, even among these studies there were important differences in study design. Some measured the BPs of the whole population,^37^ whereas others only measured the BP of patients who were normally normotensive.^39^

In comparison, six of the nine studies relating to Hurricane Katrina measured prevalence of hypertension (see table 2). Burton *et al* found the highest prevalence of hypertension among elderly enrollees on a health insurance programme (83.14%).^35^ However, the prevalence of hypertension among adults of all ages who were either in shelters or eligible for government assistance was between 25.7%^40^ and 39.5%.^41^

The studies relating to Iraq are more difficult to compare because of the different ways in which the prevalence is reported (table 3). In particular, the studies divide the prevalence into different age categories. However, as may be expected, all of the studies report that the prevalence of hypertension increases with age. For example, Doocy *et al* report a prevalence of 9.3% among adults aged 18–49 years and a prevalence of 68.8% among adults over the age of 70 years.^41 42^

For Palestinian refugees aged 40 years and above based in Jordan, Lebanon, Syrian Arab Republic, West Bank and the Gaza Strip, the prevalence of hypertension ranged from 13.8%.^43^ to 29.7%.^44^ (table 3). Most of these studies collected data from the UN Relief and Works Agency (UNRWA) primary healthcare facilities.

Again, the prevalence rates for hypertension among the Syrian studies are difficult to compare due to the different age ranges and age distributions within the study populations (table 3). However, the prevalence rates in adult populations range from 4.8%^45^ to 32%.^46^ One study considered elderly patients only, and found the rates of hypertension to be up to 83%.^47^ This population size, however, was small and the measure of hypertension was self-reported diagnosis. As with the other groups, the studies broke down prevalence rates by age, gender or location.

Looking at the remaining geographical areas, there were only four studies in the HIC group and they were all based in either the USA or Canada. Hypertension prevalence in the HIC studies varied considerably from 5.5%^48^ to 73.7%.^49^ However, this highest estimate is taken from a study with only 19 participants. Among the remaining eight LMIC studies, the prevalence ranged from 12.4%^33^ to 47%.^50^

Only four papers measured the incidence of hypertension.^31 35 49 51^ These studies all took place in HICs and incidence ranged from 10.25/1000 to 210.52/1000.

### Other outcomes

The other outcomes we examined were considered less frequently. Nine studies considered care seeking or treatment.^37 52–59^ Among studies examining utilisation of health services, a high proportion of patients with hypertension sought care for their condition in crisis settings; over 80% as reported by Rehr *et al*^56^ and Doocy *et al*^57^ (these data only relate to patients from Syria and Iraq). There was, however, no evidence detailing challenges to accessing care among patients who sought treatment.

In relation to treatment, the picture was more variable; the rates of treatment ranged from 53.4%^59^ to 98.1% of patients with hypertension.^54^ Eight studies considered BP control or medication adherence.^53 54 58–63^ This was very variable. Khader *et al*, for example, note that among Palestinian refugee patients with hypertension attending UNWRA clinics in Jordan, BP control was as high as 83%^53^ whereas Sun *et al* found that only 17.84% of patients diagnosed with hypertension after the Wenchuan earthquake had controlled BP.^59^ Sun *et al* were also the only authors to consider awareness of diagnosis.^59^

Six studies considered the barriers that patients face to manage their BP in crises, citing cost and availability of care, and severity of crisis in a given location as key reasons for disruptions in treatment and control.^60 63–67^ This group contains the only two qualitative studies included in the review. 65 66 There were also only two studies measuring what the authors considered to be a proxy for patient understanding of hypertension, that is, whether or not patients took medications as prescribed.^52 59^ This ranged from 33.1% in China to 68.5% in Iraq.

### Risk of bias

Online supplemental appendix 5 gives details of the risk of bias for each study and figure 2 summarises this across all studies. Only one of the studies was considered to be at low risk of bias.^62^ A further 10 studies were considered at low risk of bias in one domain. All other studies were at high risk of bias across both domains. Seventeen of the studies used self-reporting as the case definition, while several others did not report the case definition at all. As can be seen in tables 2 and 3, very few studies included a measure of data spread such as CIs. In addition, many of the sampling frames used were not a close representation of the target populations: for example, they may be clinic attendees, patients attending screenings or an unspecified sample of evacuation shelters.

10.1136/bmjgh-2020-002440.supp5 Supplementary data

## Discussion

This systematic review included 61 studies, 55 of which considered prevalence of hypertension. There was far less evidence relating to patient access to care and patient views of hypertension. The included studies were heterogeneous in relation to their populations and corresponding crises. It is therefore challenging to make comparisons and draw robust conclusions from the available evidence. Even studies reporting on the same crises often considered different population groups.^35 40^ In this review, the prevalence of hypertension tended to be greater among the USA and Japanese populations in comparison to the Palestinian and Syrian populations. This is in keeping with the distribution of hypertension in the general population of those countries (USA prevalence of hypertension 30% (95% CI 35%–26%), Japan 50% (95% CI 60%–42%), Palestine 16% (19%–14%) and Syria 12% (14%–10%)).^68^

Looking at the global picture, 26.4% of the adult population had hypertension in 2000 and in adults over the age of 25 years, the prevalence is estimated to be 40%.^69^ The prevalences reported in the papers we examined were generally higher than this in HIC and similar to this in LMIC (where whole population prevalences are reported). This could be due to the impact of experiencing a crisis, so called ‘disaster hypertension’ or due to overdiagnosis.^13^ It could also be due to more deprived populations at greatest risk of hypertension being situated in the most precarious areas of countries and cities.

Most of the papers included in this review did not compare the prevalence of hypertension in the crisis-affected population with that in a similar non-affected population. *Post hoc* comparison was considered using data from the Global Burden of Disease Collaborative Network.^68^ There are differences in the definition of hypertension and challenges to matching the age stratification which made this difficult.

However, one paper did compare the risk of incident hypertension in those evacuated with those not evacuated from the area affected by the Great Japan Earthquake. The authors found that in men only there was a significantly increased risk of developing hypertension in those evacuated (HR 1.20 (1.07–1.35) p=0.003).^51^ This adds further evidence to the argument that exposure to natural disasters may be an independent risk factor for the development of hypertension. However, the demography and pre-existing burden of disease in Japan means that these findings cannot be readily applied to other earthquake-prone regions such as Nepal, Pakistan and Bangladesh.

The global distribution of included studies was uneven with Japanese, American and Syrian populations being relatively well researched. This poses an information challenge for those planning services in protracted refugee situations. Eleven of the 22 recognised protracted refugee situations are in Africa and 3 are in Asia.^2^ Global skewing in different directions was also seen in other systematic reviews, although our paper is unusual in also including findings from HIC.^11 18^ Despite there being a large number of studies featured in our review, there is still a need for more widespread work to address operational needs.

As seen in other reviews examining NCDs humanitarian settings^11 18 21 22 24^ the risk of bias in the majority of included studies was high. Overall this may be due to the challenges of designing studies and collecting data in resource poor settings. In this review we found that many of the sampling frames were not representative of the target populations. In addition, while a large number of studies examined the prevalence of multiple NCDs, the data available relating to each condition were limited; for example, few studies presented information about spread of data. The reporting of degree of exposure to crises was also varied. Some studies (mainly related to natural disasters) used evacuation status as a proxy measurement for high or low exposure to an event.^70^ On the other hand, much looser measures of exposure were used in other studies, such as refugee status or country of origin in which there was a crisis.^31^

In many studies the risk of bias was high because the authors used self-reporting of hypertension or a one-off BP measurement in order to diagnose hypertension. This issue was also noted in studies of diabetes.^18^ In both cases, this results in a poor understanding of the burden of disease. Diagnosis of hypertension is getting increasingly complex. Hypertension can be conceptualised as either a disease or risk factor and this can impact on the thresholds beyond which the diagnosis is made. Further there is a discussion about how best to measure BP and when to measure it. For example, ambulatory BP measurements are a better predictor of cardiovascular disease than clinic measurements and different thresholds are recommended for diagnosis with each tool.^71^ However there is an argument that taking a risk factor approach alone, particularly at lower risk thresholds, results in overdiagnosis and overtreatment.^72^ While this is problematic in any setting, it is particularly so in crises where resources are limited and medication supplies may be erratic.

Hypertension is one of several risk factors used to predict cardiovascular outcomes of interest (usually myocardial infarction, stroke or death). In their review on the impact of armed conflict on cardiovascular disease, Jawad *et al* found that there was consistent evidence across included studies for an increase in systolic BP, mortality from chronic ischaemic heart disease and unspecified heart disease. However, there was no consistent evidence for an increase in the diagnosis of hypertension.^11^ (Although we identified papers reporting on changes in BP, these were not included as the population were not identifiable as being hypertensive or otherwise.) Among other queries, this raises the question of whether the thresholds for diagnosing hypertension are correct for this population. Prediction modelling is one approach to setting diagnostic and treatment thresholds and is dependent on the availability of longitudinal data which follows the trajectory of individuals from the time they develop the risk factor to the point at which the outcome occurs.^73^ Although WHO has produced risk prediction charts for use in different geographical areas,^74^ these do not explicitly account for the potential effect of exposure to crises.

Despite the challenges of working in humanitarian settings, several agencies have produced guidelines for the identification and management of hypertension.^75^ Attempts have been made to apply the WHO’s Package of Non-communicable Disease Interventions in humanitarian settings^76^ and the Interagency Emergency Health Kit has included a supplementary module with antihypertensive medications since 2017.^77^ Although it is unclear how widely these are being used. Furthermore, in both chronic and acute crises, psychological distress is frequently reported.^78^ Stress in its various forms increases BP and there is emerging evidence that managing stress will decrease BP.^79^ In crises, this raises the question of the optimal mode of treatment, beyond the traditional biomedical model.

This review found relatively little data collected on the cascade of care; namely patient awareness of diagnosis, seeking treatment and achieving BP control. However, in keeping with a review of diabetes care in humanitarian settings,^18^ we found that where healthcare utilisation was evaluated, high proportions of patients would seek care.^56 57^ These data are important in identifying targets for system strengthening and more details may be available in unpublished service monitoring schemes. There have been some attempts to understand barriers to care, particularly in the Middle Eastern setting. While this can help to raise awareness of potential issues in other settings, such barriers will be highly context dependent.

Control, treatment and prevention of the negative consequences of hypertension can be framed as a complex intervention. Several components are needed beyond the provision of medication. For example, dietary changes, the ability to exercise and smoking cessation efforts all form part of the ideal package of care. Those affected by humanitarian situations typically have limited control over their ability to make healthy choices. As a result, policies ranging from the choice of food parcel being funded, to the establishment of safe spaces to exercise and consideration of the ease with which tobacco can be purchased, need to be considered through a health-promotion lens.^80 81^ Both qualitative and quantitative data are needed to develop an effective offer. WHO emphasises the importance of qualitative data to inform the values, acceptability, equity and feasibility implications of its guidelines.^82^ In particular qualitative research is critical in informing the implementation of evidence into practice^83^ if we do not know the meanings that patients attach to hypertension, we cannot hope to influence their behaviour or to develop services which will achieve penetration. Lastly, both qualitative and quantitative data are needed to adequately evaluate complex interventions.

At this time, the commitment to UHC, the global epidemiological transition and the changing demography of refugee populations has come together to focus the attention of WHO, UN and NGOs working in the health sphere on NCDs. As these agencies are developing and refining guidelines, it is critical that good quality qualitative and quantitative data be generated. Our review demonstrates a particular gap in qualitative and longitudinal research.

### Strengths and limitations

There were several strengths to this review. Steps were taken to identify both published and grey literature which is important in these settings. A rigorous systematic approach was taken in the analysis and extraction of data.

There are also a number of limitations to this study. This review only considers studies published from 1 January 1999. This decision was made in order to provide an accurate estimate of the recent picture of hypertension and related health services. It does not, however, examine the effects of crises on subsequent peace-time generations. In addition, two studies could not be accessed for full-text screening.

While there were data available in some of the excluded studies that would have been relevant to the present study, the reporting of these data meant that the studies were not eligible for inclusion. Several studies that were excluded after full-text screening, for example, collected mixed data with adults and children and groups of refugees from multiple destinations. The results were reported in such a way that the groups could not be distinguished from each other and therefore the studies were excluded from the final synthesis.

Although several excluded studies did present qualitative outcomes, such as patient understanding of hypertension, and barriers and facilitators to accessing care, most of these were considered in the context of all patients with NCDs. As such the authors could not distinguish the patients with hypertension from those with other diseases. While some of these findings may have been relevant to populations with hypertension, they were not specific.

Finally, while the risk of bias tool used was appropriate for the study designs included, it did not distinguish between varying degrees of high risk of bias. Some studies, for instance, in the high risk of bias group used more robust methods than others.

## Conclusion

This study supports previous scholarship in identifying the large burden of hypertension among populations affected by humanitarian crises. This suggests that service providers going into such scenarios should plan to see and treat hypertension. Further well-conducted and reported studies are needed to examine accurate prevalence of hypertension in specific regions and also to consider the cascade of care for patients with hypertension, patient knowledge and understanding of hypertension in order to better inform service provision for this large vulnerable patient group. Longitudinal studies may provide insight into the appropriate diagnostic and treatment thresholds for hypertension in prolonged crises.
