# Supplementary material for: A systematic review of the burden of hypertension, access to services and patient views of hypertension in humanitarian crisis settings
Source: BMJ Glob Health. 2020 Nov 9;5(11):e002440. doi: 10.1136/bmjgh-2020-002440 (PMC7654140; doi:10.1136/bmjgh-2020-002440)
Supplement: Supplementary data [file bmjgh-2020-002440supp001.pdf]

19 Aug 19 - 12:01

HDAS Export

Strategy prim prev htn

See full search strategy

Strategy 699745/67

#	Database	Search term	Results
67	Medline	(exp DISASTERS/ OR (Mass Casualty Event).ti,ab OR exp REFUGEES/ OR exp "WARFARE AND ARMED CONFLICTS"/ OR exp "NATURAL DISASTERS"/ OR exp AVALANCHES/ OR exp EARTHQUAKES/ OR exp FLOODS/ OR exp LANDSLIDES/ OR exp "TIDAL WAVES"/ OR exp TSUNAMIS/ OR exp "CYCLONIC STORMS"/ OR exp DROUGHTS/ OR exp STARVATION/ OR (humanitarian ADJ2 crisis).ti,ab OR (humanitarian ADJ2 crises).ti,ab OR (humanitarian ADJ2 emergenc*).ti,ab OR (humanitarian ADJ2 relief).ti,ab OR (humanitarian ADJ2 response).ti,ab OR (humanitarian ADJ2 agenc*).ti,ab OR (refugee*).ti,ab OR (asylum seeker*).ti,ab OR (evacuee*).ti,ab OR (evacuated*).ti,ab OR (war OR wars OR warfare).ti,ab OR (avalanche*).ti,ab OR (earthquake*).ti,ab OR (flood*).ti,ab OR (landslide*).ti,ab OR (tidal wave*).ti,ab OR (tsunami*).ti,ab OR (cyclon*).ti,ab OR	9444

(hurricane*).ti,ab OR (typhoon*).ti,ab OR (drought*).ti,ab OR (famine*).ti,ab OR (starvation*).ti,ab OR (displace* ADJ2 force*).ti,ab OR (displace* ADJ2 population*).ti,ab OR (displace* ADJ2 human).ti,ab OR (displace* ADJ2 internal*).ti,ab OR (displace* ADJ2 person*).ti,ab OR (displace* ADJ2 people*).ti,ab OR (conflict* ADJ2 armed).ti,ab OR (conflict* ADJ2 zone*).ti,ab OR (conflict affected ADJ3 population*).ti,ab OR (conflict affected ADJ3 person*).ti,ab OR (conflict affected ADJ3 people*).ti,ab OR (conflict affected ADJ3 communit*).ti,ab OR (disaster* ADJ3 natural).ti,ab OR (disaster* ADJ3 victim*).ti,ab OR (disaster* ADJ3 plan*).ti,ab OR (disaster* ADJ3 relief*).ti,ab OR (persecution).ti,ab) AND (exp "ESSENTIAL HYPERTENSION"/ OR exp HYPERTENSION/ OR exp "NONCOMMUNICABLE DISEASES"/ OR (high blood pressure).ti,ab OR (HBP).ti,ab OR (HTN).ti,ab OR (NCD).ti,ab OR (non*communicable disease*).ti,ab OR (chronic diseas*).ti,ab OR (chronic condition*).ti,ab OR (long term condition*).ti,ab OR (hyperten*).ti,ab OR (cardiovascular disease).ti,ab OR (heart disease).ti,ab OR exp "BLOOD PRESSURE"/ OR (blood pressure).ti,ab OR exp "CHRONIC DISEASE"/)

(47 AND 66) [DT TO 1999]	View Results (3,018)
(47 AND 66) [DT 2000-2010]	View Results (2,666)
(47 AND 66) [DT FROM 2011]	Viewing (3,760)

19 Aug 19 - 12:32

HDAS Export
Strategy htn embase

See full search strategy

Strategy 700592/177

#	Database	Search term	Results
177	EMBASE	((humanitarian ADJ2 crisis).ti,ab OR (humanitarian ADJ2 crises).ti,ab OR (humanitarian ADJ2 emergenc*).ti,ab OR (humanitarian ADJ2 relief).ti,ab OR (humanitarian ADJ2 response).ti,ab OR (humanitarian ADJ2 agenc*).ti,ab OR (refugee*).ti,ab OR (asylum seeker*).ti,ab OR (evacuee*).ti,ab OR (evacuated*).ti,ab OR (war OR wars OR warfare).ti,ab OR (avalanche*).ti,ab OR (earthquake*).ti,ab OR (flood*).ti,ab OR (landslide*).ti,ab OR (tidal wave*).ti,ab OR (tsunami*).ti,ab OR (cyclon*).ti,ab OR (hurricane*).ti,ab OR (typhoon*).ti,ab OR (drought*).ti,ab OR (famine*).ti,ab OR (starvation*).ti,ab OR (displace* ADJ2 force*).ti,ab OR (displace* ADJ2 population*).ti,ab OR (displace* ADJ2 human).ti,ab OR (displace* ADJ2 internal*).ti,ab OR (displace* ADJ2 person*).ti,ab OR (displace* ADJ2 people*).ti,ab OR (conflict* ADJ2 armed).ti,ab OR (conflict* ADJ2 zone*).ti,ab OR	5542

(conflict affected ADJ3 population*).ti,ab OR (conflict affected ADJ3 person*).ti,ab OR (conflict affected ADJ3 people*).ti,ab OR (conflict affected ADJ3 communit*).ti,ab OR (disaster* ADJ3 natural).ti,ab OR (disaster* ADJ3 victim*).ti,ab OR (disaster* ADJ3 plan*).ti,ab OR (disaster* ADJ3 relief*).ti,ab OR (persecution).ti,ab OR exp DISASTER/ OR exp REFUGEE/ OR exp WARFARE/ OR exp WAR/ OR exp "NATURAL DISASTER"/ OR exp AVALANCHE/ OR exp EARTHQUAKE/ OR exp FLOODING/ OR exp LANDSLIDE/ OR exp TSUNAMI/ OR exp HURRICANE/ OR exp DROUGHT/ OR exp STARVATION/ OR exp HUNGER/) AND (exp "ESSENTIAL HYPERTENSION"/ OR exp HYPERTENSION/ OR exp "NONCOMMUNICABLE DISEASES"/ OR (high blood pressure).ti,ab OR (HBP).ti,ab OR (HTN).ti,ab OR (NCD).ti,ab OR (non*communicable disease*).ti,ab OR (chronic diseas*).ti,ab OR (chronic condition*).ti,ab OR (long term condition*).ti,ab OR (hyperten*).ti,ab OR (cardiovascular disease).ti,ab OR (heart disease).ti,ab OR exp "BLOOD PRESSURE"/ OR (blood pressure).ti,ab OR exp "CHRONIC DISEASE"/)

(159 AND 176) [DT TO 1998] View Results (665)

(159 AND 176) [DT 1999-2009] View Results (1,384)

(159 AND 176) [DT FROM 2010] Viewing (3,484)

19 Aug 19 - 13:10

HDAS Export
Strategy htn psych inf

See full search strategy

Strategy 702821/73

#	Database	Search term	Results
73	PsycINFO	(exp DISASTERS/ OR exp "NATURAL DISASTERS"/ OR exp REFUGEES/ OR exp WAR/ OR exp STARVATION/ OR (humanitarian ADJ2 crisis).ti,ab OR (humanitarian ADJ2 crises).ti,ab OR (humanitarian ADJ2 emergenc*).ti,ab OR (humanitarian ADJ2 relief).ti,ab OR (humanitarian ADJ2 response).ti,ab OR (humanitarian ADJ2 agenc*).ti,ab OR (refugee*).ti,ab OR (asylum seeker*).ti,ab OR (evacuee*).ti,ab OR (evacuated*).ti,ab OR (war OR wars OR warfare).ti,ab OR (avalanche*).ti,ab OR (earthquake*).ti,ab OR (flood*).ti,ab OR (landslide*).ti,ab OR (tidal wave*).ti,ab OR (tsunami*).ti,ab OR (cyclon*).ti,ab OR (hurricane*).ti,ab OR (typhoon*).ti,ab OR	988

(drought*).ti,ab OR (famine*).ti,ab OR (starvation*).ti,ab OR (displace* ADJ2 force*).ti,ab OR (displace* ADJ2 population*).ti,ab OR (displace* ADJ2 human).ti,ab OR (displace* ADJ2 internal*).ti,ab OR (displace* ADJ2 person*).ti,ab OR (displace* ADJ2 people*).ti,ab OR (conflict* ADJ2 armed).ti,ab OR (conflict* ADJ2 zone*).ti,ab OR (conflict affected ADJ3 population*).ti,ab OR (conflict affected ADJ3 person*).ti,ab OR (conflict affected ADJ3 people*).ti,ab OR (conflict affected ADJ3 communit*).ti,ab OR (disaster* ADJ3 natural).ti,ab OR (disaster* ADJ3 victim*).ti,ab OR (disaster* ADJ3 plan*).ti,ab OR (disaster* ADJ3 relief*).ti,ab OR (persecution).ti,ab) AND (exp HYPERTENSION/ OR exp "ESSENTIAL HYPERTENSION"/ OR exp "BLOOD PRESSURE"/ OR exp "BLOOD PRESSURE DISORDERS"/ OR (high blood pressure).ti,ab OR (HBP).ti,ab OR (HTN).ti,ab OR (NCD).ti,ab OR (non*communicable disease*).ti,ab OR (chronic diseas*).ti,ab OR (chronic condition*).ti,ab OR (long term condition*).ti,ab OR (hyperten*).ti,ab OR (cardiovascular disease).ti,ab OR (heart disease).ti,ab OR (blood pressure).ti,ab)

(45 AND 72) [DT FROM 2010] Viewing (538)

19 Aug 19 - 13:36

HDAS Export

Strategy htn cinahl

See full search strategy

Strategy 702832/102

#	Database	Search term	Results
102	CINAHL	(exp DISASTERS/ OR exp "MASS CASUALTY INCIDENTS"/ OR exp "NATURAL DISASTERS"/ OR exp REFUGEES/ OR exp WAR/ OR exp STARVATION/ OR (humanitarian ADJ2 crisis).ti,ab OR (humanitarian ADJ2 crises).ti,ab OR (humanitarian ADJ2 emergenc*).ti,ab OR (humanitarian ADJ2 relief).ti,ab OR (humanitarian ADJ2 response).ti,ab OR (humanitarian ADJ2 agenc*).ti,ab OR (refugee*).ti,ab OR (asylum seeker*).ti,ab OR (evacuee*).ti,ab OR (evacuated*).ti,ab OR (war OR wars OR warfare).ti,ab OR (avalanche*).ti,ab OR (earthquake*).ti,ab OR (flood*).ti,ab OR (landslide*).ti,ab OR (tidal wave*).ti,ab OR (tsunami*).ti,ab OR (cyclon*).ti,ab OR (hurricane*).ti,ab OR (typhoon*).ti,ab OR (drought*).ti,ab OR (famine*).ti,ab OR (starvation*).ti,ab OR (displace* ADJ2 force*).ti,ab OR (displace* ADJ2 population*).ti,ab OR (displace* ADJ2 human).ti,ab OR	1591

(displace* ADJ2 internal*).ti,ab OR (displace* ADJ2 person*).ti,ab OR (displace* ADJ2 people*).ti,ab OR (conflict* ADJ2 armed).ti,ab OR (conflict* ADJ2 zone*).ti,ab OR (conflict affected ADJ3 population*).ti,ab OR (conflict affected ADJ3 person*).ti,ab OR (conflict affected ADJ3 people*).ti,ab OR (conflict affected ADJ3 communit*).ti,ab OR (disaster* ADJ3 natural).ti,ab OR (disaster* ADJ3 victim*).ti,ab OR (disaster* ADJ3 plan*).ti,ab OR (disaster* ADJ3 relief*).ti,ab OR (persecution).ti,ab) AND (exp HYPERTENSION/ OR exp "ESSENTIAL HYPERTENSION"/ OR exp "NONCOMMUNICABLE DISEASES"/ OR exp "CHRONIC DISEASE"/ OR exp "BLOOD PRESSURE"/ OR (high blood pressure).ti,ab OR (HBP).ti,ab OR (HTN).ti,ab OR (NCD).ti,ab OR (non*communicable disease*).ti,ab OR (chronic diseas*).ti,ab OR (chronic condition*).ti,ab OR (long term condition*).ti,ab OR (hyperten*).ti,ab OR (cardiovascular disease).ti,ab OR (heart disease).ti,ab OR (blood pressure).ti,ab)

(85 AND 101) [DT FROM 2010]   Viewing (964)

Proquest IBSS Search Strategy

Set#: S3

Searched for: MAINSUBJECT.EXACT("Refugees") OR MAINSUBJECT.EXACT("Landslides & mudslides") OR MAINSUBJECT.EXACT("Floods") OR MAINSUBJECT.EXACT("Tidal waves") OR MAINSUBJECT.EXACT("Earthquakes") OR MAINSUBJECT.EXACT("Disasters") OR MAINSUBJECT.EXACT("Aftershocks") OR MAINSUBJECT.EXACT("Avalanches") OR MAINSUBJECT.EXACT("Tsunamis") OR OR MAINSUBJECT.EXACT("War") OR MAINSUBJECT.EXACT("Starvation") OR MAINSUBJECT.EXACT("Famine") OR MAINSUBJECT.EXACT("Hunger")

Databases: International Bibliography of the Social Sciences (IBSS)

Results: 69165

Set#: S6

Searched for: ab(humanitarian work) OR ab(humanitarian worker) OR ab(humanitarian crisis) OR ab(humanitarian aid) OR ab(humanitarian intervention) OR ab(disaster) OR ab(mass casualty events) OR ab(refugee*) OR ab(asylum seek*) OR ab(evacuee*)

Databases: International Bibliography of the Social Sciences (IBSS)

Results: 24649

Set#: S7

Searched for: ab(evacuated*) OR ab(internal displaced person) OR ab("conflict affected") OR ab(persecution) OR ab(war) OR ab(wars) OR ab(warfare) OR ab("armed conflict") OR ab(avalanche*) OR ab(earthquake*)

Databases: International Bibliography of the Social Sciences (IBSS)

Results: 103513

Set#: S8

Searched for: ab(flood*) OR ab(landslide*) OR ab("tidal wave*") OR ab(tsunami*) OR ab("cyclonic storm") OR ab(hurricane*) OR ab(drought*`) OR ab(famine*) OR ab(starvation)

Databases: International Bibliography of the Social Sciences (IBSS)

Results: 12854

Set#: S9

Searched for: S3 OR S6 OR S7 Or S8

Databases: International Bibliography of the Social Sciences (IBSS)

These databases are searched for part of your query.

Results: 169520

Set#: S10

Searched for: MAINSUBJECT.EXACT("Chronic illnesses") OR MAINSUBJECT.EXACT("Blood pressure") OR MAINSUBJECT.EXACT("Hypertension")

Databases: International Bibliography of the Social Sciences (IBSS)

Results: 1982

Set#: S11

Searched for: ab("high blood pressure") OR ab("HBP") OR ab("HTN") OR ab(hyperten*) OR ab(non-communicable disease*) OR ab("NCD*") OR ab("chronic disease*") OR ab("chronic condition*") OR ab("long term condition*")

Databases: International Bibliography of the Social Sciences (IBSS)

Results: 3791

Set#: S12

Searched for: ab("cardiovascular disease") OR ab("heart disease") OR ab("blood pressure")

Databases: International Bibliography of the Social Sciences (IBSS)

Results: 2456

Set#: S13

Searched for: s10 OR s11 OR s12

Databases: International Bibliography of the Social Sciences (IBSS)

These databases are searched for part of your query.

Results: 6324

Set#: S14

Searched for: s9 AND s13

Databases: International Bibliography of the Social Sciences (IBSS)

These databases are searched for part of your query.

Results: 133

Web of Science

Set	Results	Save History / Create Alert Open Saved History	Edit Sets	Combine Sets AND OR Combine	Delete Sets Select All Delete
# 3	2,996	#2 AND #1 *Indexes=SCI-EXPANDED, SSCI, A&HCI, CPCI-S, CPCI-SSH, ESCI Timespan=All years*	Edit		
# 2	1,014,576	**TOPIC:** (("high blood pressure") OR ("HBP") OR ("HTN") OR (hyperten*) OR (non-communicable disease*) OR ("NCD*") OR ("chronic disease*") OR ("chronic condition*") OR ("long term condition*") OR ("cardiovascular disease") OR ("heart disease") OR ("blood pressure")) *Indexes=SCI-EXPANDED, SSCI, A&HCI, CPCI-S, CPCI-SSH, ESCI Timespan=All years*	Edit		
# 1	814,010	**TOPIC:** ((humanitarian work) OR (humanitarian worker) OR (humanitarian crisis) OR (humanitarian aid) OR (humanitarian intervention) OR (disaster) OR (mass casualty event*) OR (refugee*) OR (asylum seek*) OR (evacuee*) OR (evacuated*) OR (internal displaced person) OR ("conflict affected") OR (persecution) OR (war) OR (wars) OR (warfare) OR ("armed conflict") OR (avalanche*) OR (earthquake*) OR (flood*) OR (landslide*) OR ("tidal wave*") OR (tsunami*) OR ("cyclonic storm") OR (hurricane*) OR (drought*) OR (famine*) OR (starvation)) *Indexes=SCI-EXPANDED, SSCI, A&HCI, CPCI-S, CPCI-SSH, ESCI Timespan=All years*			

Cochrane

#1 MeSH descriptor: [Natural Disasters] explode all trees 52

#2

MeSH descriptor: [Mass Casualty Incidents] explode all trees

MeSH

21

#3

MeSH descriptor: [Refugees] explode all trees

MeSH

100

#4

MeSH descriptor: [Armed Conflicts] explode all trees

MeSH

127

#5

MeSH descriptor: [Avalanches] explode all trees

MeSH

1

#6

MeSH descriptor: [Earthquakes] explode all trees

MeSH

33

#7

MeSH descriptor: [Floods] explode all trees

MeSH

3

#8

MeSH descriptor: [Landslides] explode all trees

MeSH

0

#9

MeSH descriptor: [Tidal Waves] explode all trees

MeSH

2

#10

MeSH descriptor: [Tsunamis] explode all trees

MeSH

2

#11

MeSH descriptor: [Cyclonic Storms] explode all trees

MeSH

6

#12

MeSH descriptor: [Droughts] explode all trees

MeSH

4

#13

MeSH descriptor: [Starvation] explode all trees

MeSH

47

#14

MeSH descriptor: [Relief Work] explode all trees

MeSH

63

#15

{OR #1-#14}

Limits

409

#16

((humanitarian work) OR (humanitarian worker) OR (humanitarian crisis) OR (humanitarian aid) OR (humanitarian intervention) OR (disaster) OR (mass casualty event*) OR (refugee*) OR (asylum seek*) OR (evacuee*) OR (evacuated*) OR (internal displaced person) OR ("conflict affected") OR (persecution) OR (war) OR (wars) OR (warfare) OR ("armed conflict") OR (avalanche*) OR (earthquake*) OR (flood*) OR (landslide*) OR ("tidal wave*") OR (tsunami*) OR ("cyclonic storm") OR (hurricane*) OR (drought*) OR (famine*) OR (starvation)):ti,ab,kw

S Limits

2341

#17

#15 OR #16

Limits

2424

#18

MeSH descriptor: [Hypertension] explode all trees

MeSH

16744

#19

MeSH descriptor: [Essential Hypertension] explode all trees

MeSH

135

#20

MeSH descriptor: [Noncommunicable Diseases] explode all trees

MeSH

5

#21

MeSH descriptor: [Chronic Disease] explode all trees

MeSH

12612

#22

MeSH descriptor: [Blood Pressure] explode all trees

MeSH

26751

#23

{OR #18-#22}

Limits

47340

#24

((("high blood pressure") OR ("HBP") OR ("HTN") OR (hyperten*) OR (non-communicable disease*) OR ("NCD*") OR ("chronic disease*") OR ("chronic condition*") OR ("long term condition*") OR ("cardiovascular disease") OR ("heart disease") OR ("blood pressure"))):ti,ab,kw

S Limits

157027

#25

#23 OR #24

Limits

157463

#26

#17 AND #25

Limits

154

Total = 20778

After deduplication:
