# Supplementary material for: A systematic review of the burden of hypertension, access to services and patient views of hypertension in humanitarian crisis settings
Source: BMJ Glob Health. 2020 Nov 9;5(11):e002440. doi: 10.1136/bmjgh-2020-002440 (PMC7654140; doi:10.1136/bmjgh-2020-002440)
Supplement: Supplementary data [file bmjgh-2020-002440supp002.pdf]

Appendix 2: Grey literature search strategy

ReliefWeb, UNHCR, WHO, WHO IRIS, UNICEF, MSF, IRC, ICRC, CDC, ALNAP, and Google.

("humanitarian crisis" OR warfare OR war OR “armed conflict” OR "natural disaster" OR "complex humanitarian emergency" OR refugees) AND (hypertension OR"high blood pressure" OR "blood pressure") filetype:pdf

("humanitarian crisis" OR warfare OR war OR “armed conflict” OR "natural disaster" OR "complex humanitarian emergency" OR refugees) AND (hypertension OR"high blood pressure" OR "blood pressure") filetype:pdf site:https://reliefweb.int

("humanitarian crisis" OR warfare OR war OR “armed conflict” OR "natural disaster" OR "complex humanitarian emergency" OR refugees) AND (hypertension OR"high blood pressure" OR "blood pressure") filetype:pdf site:https://www.unhcr.org

("humanitarian crisis" OR warfare OR war OR “armed conflict” OR "natural disaster" OR "complex humanitarian emergency" OR refugees) AND (hypertension OR"high blood pressure" OR "blood pressure") filetype:pdf site:https://who.int

("humanitarian crisis" OR warfare OR war OR “armed conflict” OR "natural disaster" OR "complex humanitarian emergency" OR refugees) AND (hypertension OR"high blood pressure" OR "blood pressure") filetype:pdf site:https://unicef.org

("humanitarian crisis" OR warfare OR war OR “armed conflict” OR "natural disaster" OR "complex humanitarian emergency" OR refugees) AND (hypertension OR"high blood pressure" OR "blood pressure") filetype:pdf site:https://msf.org

("humanitarian crisis" OR warfare OR war OR “armed conflict” OR "natural disaster" OR "complex humanitarian emergency" OR refugees) AND (hypertension OR"high blood pressure" OR "blood pressure") filetype:pdf site:https://rescue.org

("humanitarian crisis" OR warfare OR war OR “armed conflict” OR "natural disaster" OR "complex humanitarian emergency" OR refugees) AND (hypertension OR"high blood pressure" OR "blood pressure") filetype:pdf site:https://icrc.org

("humanitarian crisis" OR warfare OR war OR “armed conflict” OR "natural disaster" OR "complex humanitarian emergency" OR refugees) AND (hypertension OR"high blood pressure" OR "blood pressure") filetype:pdf site:https://cdc.gov

("humanitarian crisis" OR warfare OR war OR “armed conflict” OR "natural disaster" OR "complex humanitarian emergency" OR refugees) AND (hypertension OR"high blood pressure" OR "blood pressure") filetype:pdf site:https://alnap.org
