# Supplementary material for: A systematic review of the burden of hypertension, access to services and patient views of hypertension in humanitarian crisis settings
Source: BMJ Glob Health. 2020 Nov 9;5(11):e002440. doi: 10.1136/bmjgh-2020-002440 (PMC7654140; doi:10.1136/bmjgh-2020-002440)
Supplement: Supplementary data [file bmjgh-2020-002440supp003.pdf]

Appendix Three:

Articles retrieved from the data base searches excluded at the full text stage

Authors and Title	Year	Reason for exclusion
Adel: San Antonio refugees: Their demographics, healthcare profiles, and how to better serve them	2019	Cannot differentiate populations
Adu-Boahene: Health-Needs Assessment for West African Immigrants in Greater Providence, RI	2017	Cannot differentiate populations
Al Alawneh: Pharmacists in humanitarian crisis settings: Assessing the impact of pharmacist-delivered home medication management review service to Syrian refugees in Jordan	2019	Wrong article type
Amini: Quality of life in the Iranian Blind War Survivors in 2007: a cross-sectional study	2010	Study population selected on the basis of another disease
Anon: Chronic disease post Hurricane Katrina	2016	Cannot access
Anon: Launching a national surveillance system after an earthquake -- Haiti, 2010	2010	Wrong article type
Aoki: Effect of the Great East Japan Earthquake on Cardiovascular Diseases	2013	No BP measured
Arrieta: Insuring continuity of care for chronic disease patients after a disaster: key preparedness elements	2008	No BP measured
Asgary: Communicable and Non-Communicable Diseases Among Recent Immigrants with Implications for Primary care; a Comprehensive Immigrant Health Approach	2011	Cannot differentiate populations
Ay: The Perceived Barriers of Access to Health Care Among a Group of Non-camp Syrian Refugees in Jordan	2016	No BP measured
Ayappa: The Association between Health Conditions in World Trade Center Responders and Sleep-Related Quality of Life and Sleep Complaints	2019	No BP measured
Baggett: Florida disasters and chronic disease conditions	2006	Wrong article type
Bani Hani: Heart Disease in Adult Syrian Refugees: Experience at Jordan University Hospital	2019	Study population selected on the basis of another disease
Bardenheier: Trends in Chronic Diseases Reported by Refugees Originating from Burma Resettling to the United States from Camps Versus Urban Areas During 2009-2016	2019	Conflict out of date range
Barnett: Spectrum of Illness in International Migrants Seen at GeoSentinel Clinics in 1997-2009, Part 1: US-Bound Migrants Evaluated by Comprehensive Protocol-Based Health Assessment	2013	Cannot differentiate populations
Basu: Reducing chronic disease through changes in food aid: A microsimulation of nutrition and cardiometabolic disease among Palestinian refugees in the Middle East	2018	Wrong article type
Baydoun: Hurricane katrina and acute myocardial infarction: Ten years after the storm	2016	Wrong article type
Bayraktar: Elderly people as vulnerable group in disasters	2017	Wrong article type

Beiser: The health of immigrants and refugees in Canada	2005	Wrong artcle type
Beldjebel: Infectious diseases among Iraqi refugees in Lebanon	2012	Wrong artcle type
Beldjebel: Infectious diseases among refugees in Beirut	2012	Wrong artcle type
Beldjebel: Infectious diseases in refugees coming from Syria and Iraq to Lebanon	2014	Wrong artcle type
Benca: Infection among displaced population Port au Prince: Comparison of early post quake and year after earthquake periods	2011	Wrong artcle type
Bertelsen: Primary Care Screening Methods and Outcomes for Asylum Seekers in New York City	2018	Blood pressure measured but no diagnosis of hypertension
Berthold: Comorbid mental and physical health and health access in Cambodian refugees in the US	2014	Population not directly affected
Bhatta: Chronic Disease Burden Among Bhutanese Refugee Women Aged 18-65 Years Resettled in Northeast Ohio, United States, 2008-2011	2015	Conflict out of date range
Bhoopathy: Cardiovascular risk factors among men living in an internally displaced persons camp in Kenya	2014	Wrong artcle type
Bich: Impacts of flood on health: epidemiologic evidence from Hanoi, Vietnam	2011	Cannot differentiate populations
Bischoff: Health and ill health of asylum seekers in Switzerland: an epidemiological study	2009	No BP measured
Borgschulte: Health care provision for refugees in Germany - one-year evaluation of an outpatient clinic in an urban emergency accommodation	2018	Cannot differentiate populations
Boulle: Cardiovascular Disease among Syrian refugees: a descriptive study of patients in two Médecins Sans Frontières clinics in northern Lebanon	2019	Study population selected on the basis of another disease
Brackbill: Physical health consequences of being injured during the world trade center terrorist attacks on september 11, 2001	2013	Study population selected on the basis of another disease
Burns: Health status of returning refugees, internally displaced persons, and the host community in a post-conflict district in northern Sri Lanka: a cross-sectional survey	2018	No BP measured
Byberg: Cardiovascular disease incidence and survival: Are migrants always worse off?	2016	No BP measured
Bydzovsky: Experience with migrants on Balkan Route from the Field Hospital on the Slovenian-Croatian Border	2016	Cannot differentiate populations
Centers for Disease, Control and Prevention: Assessment of health-related needs after Hurricanes Katrina and Rita--Orleans and Jefferson Parishes, New Orleans area, Louisiana, October 17-22, 2005	2006	No BP measured
Centers for Disease, Control and Prevention: Illness surveillance and rapid needs assessment among Hurricane Katrina evacuees--Colorado, September 1-23, 2005	2006	No BP measured
Centers for Disease, Control and Prevention: Morbidity surveillance after Hurricane Katrina--Arkansas, Louisiana, Mississippi, and Texas, September 2005	2006	No BP measured

Centers for Disease, Control and Prevention: Rapid assessment of health needs and resettlement plans among Hurricane Katrina evacuees--San Antonio, Texas, September 2005	2006	No BP measured
Centers for Disease, Control and Prevention: Rapid assessment of the needs and health status of older adults after Hurricane Charley--Charlotte, DeSoto, and Hardee Counties, Florida, August 27-31, 2004	2004	No BP measured
Chaaban: Recent changes in welfare indicators among Palestinian refugees in Lebanon: a comparative study of two cross-sectional datasets	2019	Wrong artcle type
Chan: Characteristics and health outcomes of internally displaced population in unofficial rural self-settled camps after the 2005 Kashmir, Pakistan earthquake	2010	No BP measured
Chan: Chronic health needs immediately after natural disasters in middle-income countries: the case of the 2008 Sichuan, China earthquake	2011	Cannot differentiate populations
Chan: Chronic health needs immediately after natural disasters in middle-income countries: the case of the 2008 Sichuan, China earthquake	2010	Cannot differentiate populations
Chan: Comparision of health needs of older people between affected rural and urban areas after the 2005 Kashmir, Pakistan earthquake	2009	No BP measured
Chandra: Prevalence of chronic disease risk factors in 35- to 44-year-old humanitarian arrivals to New South Wales (NSW), Australia	2019	Cannot differentiate populations
Charles-Larco: Prise en charge des patients diabetiques apres le seisme du 12 janvier 2010 en HaitiManagement of diabetic patients after the earthquake in Haiti January 12, 2010	2011	Wrong article type
Chen: Acute cardiovascular effects of the Wenchuan earthquake: ambulatory blood pressure monitoring of hypertensive patients	2009	Blood pressure measured but no diagnosis of hypertension
Chen: Emotions of depression and anxiety from earthquake have significant influence on diabetes morbidity at the disaster areas after 2008 5.12 sichuan earthquake	2010	Wrong artcle type
Cho: Weight gain and the risk of metabolic syndrome among North Korean refugees living in South Korea - An example of thrifty hypothesis	2010	Wrong artcle type
Cline: The resettlement experiences of African refugee single mothers and hypertension management in United States	2014	Wrong artcle type
Collins: Cardiovascular disease risk and prevention amongst Syrian refugees: mixed methods study of MÃ©decins Sans FrontiÃ¨res programme in Jordan	2017	Blood pressure measured but no diagnosis of hypertension
Cope: Estimating the factors associated with health status and access to care among Iraqis displaced in Jordan and Syria using population assessment data	2012	Wrong artcle type
Culhane-Pera: Cardiovascular disease risks in Hmong refugees from Wat Tham Krabok, Thailand	2009	Conflict out of date range
Da Silva: Global health within borders: Outlook of health screening among Iraqi and sudanese refugee community and the impact of language barrier the patient self-advocacy	2015	Wrong artcle type

Dalmar: Living in ethnic-enclave neighborhoods may attenuate the negative effect of acculturation on blood pressure in refugees and maintain immigrant health effect	2017	Wrong article type
Damiri: Metabolic syndrome among overweight and obese adults in Palestinian refugee camps	2018	Study population selected on the basis of another disease
Darvishi: Investigating Prevalence and Pattern of Long-term Cardiovascular Disorders in Sulphur Mustard-exposed Victims and Determining Proper Biomarkers for Early Defining, Monitoring and Analysis of Patients' Feedback on Therapy	2017	Wrong article type
Deere: Incidence of acute myocardial infarction and hurricane katrina: Four-fold increase eleven years after the storm	2018	Wrong article type
Del Pinto: Health status of Afro-Asian refugees in an Italian urban area: a cross-sectional monocentric study	2018	Cannot differentiate populations
Di Castelnuovo: Effect of the l'aquila earthquake on metabolic syndrome in survivors from the 2009 earthquake in Italy	2012	Wrong article type
DiGeronimo: Impact of hurricane Irma on prescription fill patterns in a medicare population with chronic conditions	2018	Wrong article type
Donker: Chronic diseases of victims and controls before and after disaster	2005	Wrong article type
Doocy: Guidelines and mHealth to Improve Quality of Hypertension and Type 2 Diabetes Care for Vulnerable Populations in Lebanon: Longitudinal Cohort Study	2017	Cannot differentiate populations
Dookeran: Chronic disease and its risk factors among refugees and asylees in Massachusetts, 2001-2005	2010	Blood pressure measured but no diagnosis of hypertension
Dorman: Health Status of North Korean Refugees in Toronto: A Community Based Participatory Research Study	2017	Cannot differentiate populations
Dudova: Six month follow up in communicable versus non-communicable diseases in an Iraqi refugee camp	2016	Cannot differentiate populations
Ebner: Increase in metabolic diseases following the Fukushima triple disaster: A retrospective study of Kawauchi Village with long-term follow-up	2015	Wrong article type
El Arnaout: Assessment of the health needs of Syrian refugees in Lebanon and Syria's neighboring countries	2019	Wrong article type
Fahd: Non-communicable disease risk factors in Palestine refugees in Lebanon: A descriptive study	2018	Wrong article type
Feldman: Examining the impact of captivity in men and women on the slaves returning to south Sudan	2014	Wrong article type
Ferdinand: The Hurricane Katrina disaster: focus on the hypertensive patient	2005	Wrong article type
Fergus: Volcanic post traumatic stress and the impact on cardiometabolic health	2012	Wrong article type
Frost: Determining Physical and Mental Health Conditions Present in Older Adult Refugees: A Mini-Review	2019	Cannot differentiate populations
Führer: Morbidity of asylum seekers in a medium-sized German city	2016	No BP measured
Fuse: Onsite medical rounds and fact-finding activities conducted by Nippon Medical School in Miyagi prefecture after the Great East Japan Earthquake 2011	2011	No BP measured

GÃ³mez-Restrepo: Enfermedades crÃ³nicas en poblaciÃ³n afectada por el conflicto armado en Colombia, 2015	2017	Duplicate
Gallegos: Living well multicultural-does it change behaviours in migrants to reduce the risk of chronic disease?	2016	Wrong article type
Gammoh: A preliminary description of medical complaints and medication consumption among 375 Syrian refugees residing in North Jordan	2016	Cannot differentiate populations
Gammouh: Chronic diseases, lack of medications, and depression among Syrian refugees in Jordan, 2013-2014	2015	No BP measured
Gardemann: A record of morbidity and medical request profiles in international humanitarian aid, taking the earthquake in Bam in Iran in 2003 as an example	2009	Cannot differentiate populations
Gerin: Sustained blood pressure increase after an acute stressor: the effects of the 11 September 2001 attack on the New York City World Trade Center	2005	Population not directly affected
Gerritsen: [Mental and physical health problems of, and the use of healthcare by, Afghan, Iranian and Somali asylum seekers and refugees]	2006	Cannot differentiate populations
Gerritsen: Physical and mental health of Afghan, Iranian and Somali asylum seekers and refugees living in the Netherlands	2006	No BP measured
Ghattas: Poverty, food insecurity, and health of Palestinian refugees in Lebanon and recently displaced from Syria to Lebanon: Findings from the 2015 socioeconomic household survey	2018	Wrong article type
Ghosh: Rapid needs assessment among Hurricane Katrina evacuees in metro-Denver	2007	Cannot differentiate populations
Giorgini: Long-term blood pressure changes induced by the 2009 L'Aquila earthquake: assessment by 24 h ambulatory monitoring	2013	Study population selected on the basis of another disease
Golub: Impact of Length of Residence in the United States on Risk of Diabetes and Hypertension in Resettled Refugees	2018	Cannot differentiate populations
Goosen: THE RELATIONSHIP BETWEEN POST TRAUMATIC STRESS DISORDER AND HYPERTENSION AMONG 105 180 ASYLUM SEEKERS IN THE NETHERLANDS	2011	No BP measured
Gordon: Lessons Learned from a Medical Response Team 45 Days Post Hurricane Maria in Puerto Rico	2019	No BP measured
Guha-Sapir: Short communication: patterns of chronic and acute diseases after natural disasters - a study from the International Committee of the Red Cross field hospital in Banda Aceh after the 2004 Indian Ocean tsunami	2007	Cannot differentiate populations
Habib: Associations between life conditions and multi-morbidity in marginalized populations: the case of Palestinian refugees	2014	Cannot differentiate populations
Hameed: Acute myocardial infarction before and after the storm: Hurricane Katrina	2012	Wrong artcle type
Hanna: Delivering healthcare to the refugee population in Pittsburgh	2015	Wrong artcle type
Hanzawa: Below-the-knee DVT related with cerebral infarction after Mid Niigata Prefecture Earthquake 2004	2014	Wrong artcle type
Hanzawa: Hypertension is a risk factor for DVT in Japanese	2011	Wrong artcle type

quake residents		
Hanzawa: Increasing of ischemic stroke in residents with DVT after mid Niigata prefecture earthquake	2011	Wrong artcle type
Hasegawa: Emergency Responses and Health Consequences after the Fukushima Accident; Evacuation and Relocation	2016	Wrong artcle type
Hashimoto: Influence of post-disaster evacuation on incidence of metabolic syndrome	2017	Blood pressure measured but no diagnosis of hypertension
He: Acute changes of blood pressure and heart rate induced by a strong earthquake	2010	Blood pressure measured but no diagnosis of hypertension
Henry: Violence and the body: somatic expressions of trauma and vulnerability during war	2006	Wrong artcle type
Higgins: Role of the Ambulatory Care Clinical Pharmacist in Management of a Refugee Patient Population at a University-Based Refugee Healthcare Clinic	2019	Study population selected on the basis of another disease
Holman: Terrorism, acute stress, and cardiovascular health: a 3-year national study following the September 11th attacks	2008	Population not directly affected
Holt: Hurricane katrina related experiences and blood pressure control in older adults: Findings from cosmo	2012	Wrong artcle type
Horikoshi: The effect of telephone support to evacuees with risks of hypertension and diabetes mellitus after a disaster: the Fukushima Health Management Survey	2017	No BP measured
Hozawa: Tohoku medical megabank project community-based cohort study	2019	Wrong artcle type
Hu: Change characteristics of artery blood pressure in people with starvation and refeeding	2011	Wrong artcle type
Hung: Disease pattern and chronic illness in rural China: the Hong Kong Red Cross basic health clinic after 2008 Sichuan earthquake	2013	Cannot differentiate populations
Hung: Disease patterns in a rural setting three weeks after the sichuan earthquake-Hong Kong red cross basic health clinic in Yanmen town	2010	Wrong artcle type
Hussain: Burden of non-communicable diseases in Iraq after the 2003 war	2019	Cannot differentiate populations
Ishibashi: Study of headache after the great east Japan earthquake in iwate coast area (1)report of 2012	2015	Wrong artcle type
Israfilov: The distribution of arterial hypertension as risk factor of coronary disease among refugees men at 20-59 age in Sumgait City	2006	Cannot access
Ito: Transient increase in blood pressure after the Great East Japan Earthquake in patients with hypertension living around Tokyo	2013	Wrong artcle type
Jamil: Self-rated Health and Medical Conditions in Refugees and Immigrants from the Same Country of Origin	2015	Conflict out of date range
Jen: Sex differences and predictors of changes in body weight and noncommunicable diseases in a random, newly-arrived group of refugees followed for two years: Erratum	2018	Blood pressure measured but no diagnosis of hypertension
Jhung: Chronic disease and disasters medication demands of	2007	No BP measured

Hurricane Katrina evacuees		
Jiao: DID Hurricane Katrina continue to affect the incidence of acute coronary syndromes in New Orleans?	2011	Wrong artcle type
Jiao: Effect of Hurricane Katrina on incidence of acute myocardial infarction in New Orleans three years after the storm	2012	Study population selected on the basis of another disease
Jonassen: Socioeconomic status and chronic disease in Palestinians living in and outside refugee camps in the West Bank and the Gaza Strip: An observational study	2018	Wrong artcle type
Jonassen: Socio-economic status and chronic disease in the West Bank and the Gaza Strip: in and outside refugee camps	2018	Cannot differentiate populations
Joob: Tayland'daki buyuk selde, hipertansiyonlu hastalarin takibinde kayip sorunuBig flooding in thailand, the problem on loss following up of patients with hypertension	2012	Wrong artcle type
Jordan: Heart disease among adults exposed to the September 11, 2001 World Trade Center disaster: results from the World Trade Center Health Registry	2011	Cannot differentiate populations
Kamkamidze: SELF-PERCEIVED HEALTH STATUS AND ILLNESSES AMONG INTERNALLY DISPLACED PEOPLE IN GEORGIA	2018	No BP measured
Kario: Factors associated with the occurrence and magnitude of earthquake-induced increases in blood pressure	2001	Wrong artcle type
Kawasaki: The basic data for residents aged 16 years or older who received a comprehensive health check examinations in 2011-2012 as a part of the Fukushima Health Management Survey after the great East Japan earthquake	2014	Blood pressure measured but no diagnosis of hypertension
Kayitesi: Acquisition of cardiovascular disease risk factors among refugees and immigrants: A longitudinal study	2015	Wrong artcle type
Khader: Treatment outcomes in a cohort of Palestine refugees with diabetes mellitus followed through use of E-Health over 3 years in Jordan	2014	Study population selected on the basis of another disease
Khan: REFUGEE HEALTH AND REHABILITATION: CHALLENGES AND RESPONSE	2017	Wrong artcle type
Kim: Excess weight gain in nonobese North Korean refugees associated with increased risk of impaired fasting glucose	2014	Wrong artcle type
Kim: Post-Nargis medical care: experience of a Korean Disaster Relief Team in Myanmar after the cyclone	2010	No BP measured
Kim: Prevalence of metabolic syndrome and its related factors among North Korean refugees in South Korea: A cross-sectional study	2016	Cannot differentiate populations
Kim: Prevalence of metabolic syndrome and its related factors among North Korean refugees in South Korea: A cross-sectional study	2016	Conflict out of date range
Kira: D. The physical and mental status of Iraqi refugees and its etiology	2007	Cannot differentiate populations
Kloner: Lessons learned about stress and the heart after major earthquakes	2019	Wrong artcle type
Konno: Blood pressure among public employees after the Great East Japan Earthquake: the Watari study	2013	Population not directly affected
Konno: Blood pressure changes over 2 years after the great	2013	Wrong artcle type

east japan earthquake and associated cardiovascular risk factors: The watari study		
Konno: Blood Pressure Elevation Lasting Longer Than 1 Year Among Public Employees After the Great East Japan Earthquake: The Watari Study	2017	Population not directly affected
Konno: Prolonged pressor effects of the great east Japan earthquake in public employees: The watari study	2012	Wrong artcle type
Kory: Health ramifications of the Gush Katif evacuation	2013	Cannot differentiate populations
Krcmery: Early versus late onset infections during Haiti earthquake 2010	2013	Wrong artcle type
Kumar: Noninfectious disease among the Bhutanese refugee population at a United States urban clinic	2014	Conflict out of date range
Kumar: The burden of chronic disease among the bhutanese refugee population at a US Urban clinic	2011	Wrong artcle type
Kuroda: Health care response to the tsunami in Taro District, Miyako City, Iwate Prefecture	2011	No methods
Lenane: Association of Post-Traumatic Stress Disorder Symptoms Following Hurricane Katrina With Incident Cardiovascular Disease Events Among Older Adults With Hypertension	2019	No BP measured
Li: Acute effects of the YaAn earthquake on blood pressure among hospitalized patients in the department of cardiology	2014	Wrong artcle type
Li: YaAn earthquake increases blood pressure among hospitalized patients	2016	Blood pressure measured but no diagnosis of hypertension
Lipsky: World Trade Center disaster effect on blood pressure	2002	Wrong artcle type
Lobjanidze: COMPARISON OF INTERFERENCE OF HYPERTENSION AND SLEEP DISORDERS AMONG NORMAL ADULT POPULATION AND REFUGEES	2016	Wrong artcle type
Ludwig: When you are here, you have high blood pressure: Liberian refugeesâ€™ health and access to healthcare in Staten Island, NY	2016	No BP measured
MacPherson: Health of displaced Albanian Kosovars in the former Yugoslav Republic of Macedonia: fitness to travel and health outcomes assessment	2002	Cannot differentiate populations
Malik: Disease Status of Afghan Refugees and Migrants in Pakistan	2019	No BP measured
Malla: Analysis of Post Earthquake Disease Pattern in a Camp at Gyampesal Gorkha	2016	Study population selected on the basis of another disease
Mani: Relationship between particulate matter exposure and atherogenic profile in "Ground Zero" workers as shown by dynamic contrast enhanced MR imaging	2013	Military or service personnel
Markoglou: Epidemiologic characteristics of hypertension in the civilians of Kosovo after the war	2005	Cannot differentiate populations
Marshall: Diabetes and Cardiovascular Disease Risk in Cambodian Refugees	2016	Cannot differentiate populations
Massad: Metabolic syndrome among refugee women from the west bank, Palestine: A cross-sectional study	2018	Cannot differentiate populations
McEniry: Early Life Displacement Due to Armed Conflict and	2019	Wrong artcle type

Violence, Early Nutrition, and Older Adult Hypertension, Diabetes, and Obesity in the Middle-Income Country of Colombia		
McLeod: The health status of quota refugees screened by New Zealand's Auckland Public Health Service between 1995 and 2000	2005	No methods
Millin: A comparative analysis of two external health care disaster responses following Hurricane Katrina	2006	Cannot differentiate populations
Misra: Chronic Health Conditions, Physical Activity and Dietary Behaviors of Bhutanese Refugees: A Houston-Based Needs Assessment	2016	Conflict out of date range
Moran: For What Illnesses Do Asylum Seekers and Undocumented Migrant Workers in Israel Seek Healthcare? An Analysis of Medical Visits at a Large Urgent Care Clinic for the Uninsured in Tel Aviv	2019	No BP measured
Morrison: When Community Calls, We Collaborate! Community-Based Participatory Research With the Multilanguage Montagnard Refugee Community	2018	Wrong artcle type
Moscona: Increased incidence of acute coronary syndrome following hurricane Katrina in new Orleans: The impact continues	2013	Wrong artcle type
Moscona: The effects of hurricane Katrina on acute myocardial infarction five years after the storm	2012	Wrong artcle type
Moscona: The Incidence, Risk Factors, and Chronobiology of Acute Myocardial Infarction Ten Years After Hurricane Katrina	2019	Population not directly affected
Mousa: Hyperglycemie, hypertension arterielle et facteurs de risque de ces maladies chez les refugies palestiniens pris en charge par l'UNRWA<br>Hyperglycaemia, hypertension and their risk factors among Palestine refugees served by UNRWA	2010	Duplicate
Mullins: Cohort reporting improves hypertension care for refugees	2012	Wrong artcle type
Mulugeta: Burden of Mental Illness and Non-communicable Diseases and Risk Factors for Mental Illness Among Refugees in Buffalo, NY, 2004-2014	2019	Cannot differentiate populations
Murakami: A cross-sectional survey of blood pressure of a coastal city's resident victims of the 2011 Tohoku tsunami	2013	Cannot differentiate populations
Muramatsu: The course of blood pressure and diabetes and mood disorders among primary care patients after the Chuetsu earthquake in Japan	2005	Wrong artcle type
Mykhailichenko: Arterial hypertension is the key risk factor of vascular events in the conditions of war and economic blockade in the Donbass region	2017	Wrong artcle type
Nakaya: Psychological Distress and the Risk of Withdrawing From Hypertension Treatment After an Earthquake Disaster	2017	No BP measured
Neria: Mental and physical health consequences of the September 11, 2001 (9/11) attacks in primary care: a longitudinal study	2013	Population not directly affected
Neupane: CHANGE IN BLOOD PRESSURE FOLLOWING NEPAL'S EARTHQUAKE: A COMPARATIVE CROSS-SECTIONAL STUDY	2016	Wrong artcle type
Nguyen: Prevalence of Chronic Disease and Their Risk Factors Among Iranian, Ukrainian, Vietnamese Refugees in California, 2002-2011	2016	Cannot differentiate populations

Nishizawa: Salt-intake and risk of disaster hypertension among evacuees at shelter of the great East Japan earthquake	2019	Wrong artcle type
Noe: Disaster-related injuries and illnesses treated by American Red Cross disaster health services during Hurricanes Gustav and Ike	2013	Cannot differentiate populations
Ochi: The great East Japan earthquake disaster: a compilation of published literature on health needs and relief activities, march 2011-september 2012	2013	Wrong artcle type
Odunukan: Addressing the care of chronic diseases in post disaster responses	2012	Wrong artcle type
Ohira: Changes in Cardiovascular Risk Factors After the Great East Japan Earthquake	2017	Wrong artcle type
Ohira: Changes in metabolic profiles among evacuees after the great east Japan earthquake: The fukushima health management survey	2014	Wrong artcle type
Petrarca: Retrospective evaluation of blood pressure changes after the 2009 L'Aquila earthquake	2011	Wrong artcle type
Petrazzi: Changes in 24-hour ambulatory blood pressure monitoring during the 2009 earthquake at L'Aquila	2010	Wrong artcle type
Pfortmueller: Adult Asylum Seekers from the Middle East Including Syria in Central Europe: What Are Their Health Care Problems?	2016	No BP measured
Pfortmueller: Multimorbidity in adult asylum seekers: a first overview	2013	No BP measured
Qato: Medication utilization in the palestine refugee population in the middle east: A cross-country comparative analysis	2014	Wrong artcle type
Ramphal: Medical and psychosocial needs of the Puerto Rican people after Hurricane Maria	2018	No BP measured
Redditt: Health status of newly arrived refugees in Toronto, Ont: Part 2: chronic diseases	2015	Cannot differentiate populations
Reed: Investigating the Refugee Health Disadvantage Among the U.S. Immigrant Population	2017	Cannot differentiate populations
Renteria-Ramos: Morbi-Mortality of the Victims of Internal Conflict and Poor Population in the Risaralda Province, Colombia	2019	No BP measured
Renzaho: Prevalence of vitamin D insufficiency and risk factors for type 2 diabetes and cardiovascular disease among African migrant and refugee adults in Melbourne: a pilot study	2011	Cannot differentiate populations
Rhodes: Development of obesity and related diseases in a prospective longitudinal cohort of African refugees compared to matched regional controls	2015	Wrong artcle type
Rhodes: Development of Obesity and Related Diseases in African Refugees After Resettlement to United States	2016	Cannot differentiate populations
Ridenour: Displacement of the underserved: medical needs of Hurricane Katrina evacuees in West Virginia	2007	No BP measured
Russo: Health profile and disease determinants among asylum seekers: a cross-sectional retrospective study from an Italian reception centre	2016	No BP measured
Ryan: Determining Key Influences on Patient Ability to Successfully Manage Noncommunicable Disease After Natural Disaster	2019	Wrong artcle type
Saito: Increased incidence of acute myocardial infarction after the great East Japan earthquake	2012	Wrong artcle type

Salazar: Health Consequences of an Armed Conflict in Zamboanga, Philippines Using a Syndromic Surveillance Database	2018	Cannot differentiate populations
Salazar: Increased incidence of acute myocardial infarction after the great East Japan earthquake	2017	Cannot differentiate populations
Salazar: Post-disaster health impact of natural hazards in the Philippines in 2013	2016	Cannot differentiate populations
Saleh: mHealth use for non-communicable diseases care in primary health: patients' perspective from rural settings and refugee camps	2018	Wrong artcle type
Saleh: Using Mobile Health to Enhance Outcomes of Noncommunicable Diseases Care in Rural Settings and Refugee Camps: Randomized Controlled Trial	2018	Cannot differentiate populations
Sasabuchi: Effect of the 2016 Kumamoto earthquakes on preventable hospital admissions: a retrospective cohort study in Japan	2018	No BP measured
Sato: Risk Factors and Prevalence of Deep Vein Thrombosis After the 2016 Kumamoto Earthquakes	2019	Study population selected on the basis of another disease
Satoh: Acute and subacute effects of the great East Japan earthquake on home blood pressure values	2011	Wrong artcle type
Sawano: Non-communicable diseases in decontamination workers in areas affected by the Fukushima nuclear disaster: a retrospective observational study	2016	Military or service personnel
Schnall: Disaster-Related Shelter Surveillance During the Hurricane Harvey Response - Texas 2017	2019	No BP measured
Schnall: Disaster-Related Surveillance Among US Virgin Islands (USVI) Shelters During the Hurricanes Irma and Maria Response	2019	No BP measured
Schneider: Disparities in health and access to healthcare between asylum seekers and residents in Germany: a population-based cross-sectional feasibility study	2015	No BP measured
Semere: Factors Associated with Refugee Acute Healthcare Utilization in Southern Connecticut	2018	Cannot differentiate populations
Semere: Timing of refugee health assessments may be associated with acute healthcare utilization	2016	Wrong artcle type
Serre-Delcor: Health Status of Asylum Seekers, Spain	2018	No methods
Shah: Disease pattern in earthquake affected areas of Pakistan: data from Kaghan valley	2010	Cannot differentiate populations
Shahin: Diabetes care in refugee camps: the experience of UNRWA	2015	Wrong artcle type
Sharif: A Health Profile and Overview of Healthcare Experiences of Cambodian American Refugees and Immigrants Residing in Southern California	2019	Wrong artcle type
Sharma: Chronic disease and related conditions at emergency treatment facilities in the New Orleans area after Hurricane Katrina	2008	No BP measured
Shehab: Access to care among displaced Mississippi residents in FEMA travel trailer parks two years after Katrina	2008	No BP measured
Slama: Care of non-communicable diseases in emergencies	2017	Wrong artcle type
Smith: Health Conditions of Post-Resettlement African Refugees in Boise, Idaho	2016	Cannot differentiate populations

Sofia: Cardiovascular and Cerebrovascular Events Pre- and Post-Earthquake of 6 April 2009: The Abruzzo's Experience	2012	Study population selected on the basis of another disease
Strand: Short-Term Public Health Impact of the July 22, 2011, Terrorist Attacks in Norway: A Nationwide Register-Based Study	2016	No BP measured
Stratta: Effects on health of the L'Aquila (Central Italy) 2009 earthquake	2016	Wrong artcle type
Striuli: Changes in 24-h ambulatory blood pressure during the 2009 earthquake at L'Aquila: a new evaluation in the same patients	2013	Wrong artcle type
Takahashi: Impact of a major natural disaster on longitudinal changes in cardiovascular risk factors in the general population	2015	Wrong artcle type
Taleshan: Multimorbidity and mortality thereof, among non-western refugees and family reunification immigrants in Denmark - a register based cohort study	2018	No BP measured
Tamblyn: Patients from abroad becoming patients in everyday practice: torture survivors in primary care	2011	Cannot differentiate populations
Tan: Medical response to the 2009 Sumatra earthquake: Health needs in the post-disaster period	2012	No BP measured
Tanaka: Predictors of Hypertension in Survivors of the Great East Japan Earthquake, 2011: A Cross-sectional Study	2016	Cannot differentiate populations
Tanigawa: MON-336 LONGITUDINAL ANALYIS OF KIDNEY FUNCTION AND POSSIBLE FACTORS INVOLVED AFTER THE GREAT EAST JAPAN EARTHQUAKE AMONG RESIDENTS NEAR THE FUKUSHIMA DAIICHI NUCLEAR POWER PLANT	2019	Wrong artcle type
Tavilla: Blood pressure changes in African males during application for asylum status in Italy: A pilot study	2018	Wrong artcle type
Tayfur:Healthcare Service Access and Utilization among Syrian Refugees in Turkey	2019	No BP measured
Terayama: Impact of the great East Japan earthquake on stroke incidence among survivors in sanriku coastal area	2013	Wrong artcle type
Thompson: Effect of hurricane katrina on medication adherence and blood pressure among hypertensive veterans	2012	Wrong artcle type
Tiong: Health issues in newly arrived African refugees attending general practice clinics in Melbourne	2006	No BP measured
Toar: Comparison of self-reported health & healthcare utilisation between asylum seekers and refugees: an observational study	2009	No BP measured
Tomio: Interruption of medication among outpatients with chronic conditions after a flood	2010	Study population selected on the basis of another disease
Trovata: Dangerous crossing: demographic and clinical features of rescued sea migrants seen in 2014 at an outpatient clinic at Augusta Harbor, Italy	2016	Cannot differentiate populations
Truppa: Utilization of primary health care services among Syrian refugee and Lebanese women targeted by the ICRC program in Lebanon: a cross-sectional study	2019	No BP measured
Tsubokura: The immediate physical and mental health crisis in residents proximal to the evacuation zone after Japan's	2014	Blood pressure measured but no

nuclear disaster: an observational pilot study		diagnosis of hypertension
Uscher-Pines: The physical health impacts of post-disaster displacement: A study of the older adult victims of Hurricane Katrina	2008	Wrong artcle type
van Berlaer: A refugee camp in the centre of Europe: clinical characteristics of asylum seekers arriving in Brussels	2016	Cannot differentiate populations
van Melle: Quality of primary care for resettled refugees in the Netherlands with chronic mental and physical health problems: a cross-sectional analysis of medical records and interview data	2014	Cannot differentiate populations
Vukovia: Psychological and somatic health problems in Bosnian refugees: a three year follow-up	2014	Cannot differentiate populations
Vulic: Post-trauma cardiovascular risk factors and subclinical atherosclerosis in young adults following the war in Bosnia and Herzegovina	2019	Cannot differentiate populations
Vulic: Post-trauma cardiovascular risk factors and subclinical atherosclerosis in young adults following war in bosnia-herzegovina	2012	Wrong artcle type
Vulic: Post-trauma predictors cardiovascular diseaseis in young adults following war	2013	Wrong artcle type
Wagner: Trauma, healthcare access, and health outcomes among Southeast Asian refugees in Connecticut	2013	Cannot differentiate populations
Watts: Determinants of health care access on the U.S.-Mexico border	2009	Wrong artcle type
Wen: Quality of life, physical diseases, and psychological impairment among survivors 3 years after Wenchuan earthquake: a population based survey	2012	No BP measured
Wieland: Diabetes care among Somali immigrants and refugees	2012	Study population selected on the basis of another disease
Wiwanitkit: Post-earthquake chronic illness	2013	Duplicate
Wiwanitkit: Post-earthquake chronic illness...Hung KKC, Lam ECC, Chan EYY, Graham CA. Disease pattern and chronic illness in rural China: the Hong Kong Red Cross basic health clinic after 2008 Sichuan earthquake. Emerg. Med. Australas. 2013; 25: 252–259	2013	Wrong artcle type
Woersching: Post-earthquake chronic illness	2003	Cannot differentiate populations
Wu: A cross-sectional survey on the health status and the health-related quality of life of the elderly after flood disaster in Bazhong city, Sichuan, China	2015	No BP measured
Yamauchi: Impact of ethnic conflict on the nutritional status and quality of life of suburban villagers in the Solomon Islands	2010	Blood pressure measured but no diagnosis of hypertension
Yanni: The health profile and chronic diseases comorbidities of US-bound Iraqi refugees screened by the International Organization for Migration in Jordan: 2007-2009	2013	Cannot differentiate populations
Yoda: Relationship Between Long-term Flooding and Serious Mental Illness After the 2011 Flood in Thailand	2017	Cannot differentiate populations
Yun: High prevalence of chronic non-communicable conditions	2012	Cannot differentiate

among adult refugees: implications for practice and policy		populations
Yun: Prevalence of chronic disease and insurance coverage among refugees in the United States	2012	Cannot differentiate populations
Yun: Prevalence of chronic disease and insurance coverage among refugees in the United States	2012	Cannot differentiate populations
Zabaneh: Living and health conditions of Palestinian refugees in an unofficial camp in the Lebanon: a cross-sectional survey	2008	No BP measured
Zeynalov: Epidemiological features of the main risk factors for cardiovascular disease among the unorganized man's population of Baku and Sumgait city population of internally displaced persons 20-59 years	2016	Population not directly affected
Zhihong: The impact of anxiety on blood pressure of flood victims	2011	Wrong artcle type
