# Supplementary material for: A systematic review of the burden of hypertension, access to services and patient views of hypertension in humanitarian crisis settings
Source: BMJ Glob Health. 2020 Nov 9;5(11):e002440. doi: 10.1136/bmjgh-2020-002440 (PMC7654140; doi:10.1136/bmjgh-2020-002440)
Supplement: Supplementary data [file bmjgh-2020-002440supp004.pdf]

Appendix Four:

Articles retrieved from the grey literature searches excluded at the full text stage

Authors and Title	Year	Reason
A Qualitative Cross-sectional Cluster Survey: Population-Based Health Access Assessment for Syrian Refugees in Non-Camp Settings Throughout Jordan		No BP measured
Al-rousan: Health needs and priorities of Syrian refugees in camps and urban settings in Jordan : perspectives of refugees and health care providers	2018	Qualitative study talking to general population
Al-sharafi: Endocrinology & Metabolic Syndrome The Effect of War on the Control of Diabetes in Patients with Type 2 Diabetes	2017	Study population selected on the basis of another disease
Amara: Health needs and priorities of Syrian refugees in camps and urban settings in Jordan : perspectives of refugees and health care providers	2014	Wrong article type
American Near East Refugee Aid: Palestinian Refugees from Syria in Lebanon	2013	Cannot differentiate populations
Anon: HEALTH Chapter		Wrong article type
Arnaout: Assessment of the health needs of Syrian refugees in Lebanon and Syria's neighboring countries	2019	Wrong article type
Bhatiasevi: Humanitarian crisis after the Nepal earthquakes 2015	2015	No methods
Boulle: Cardiovascular Disease among Syrian refugees : a descriptive study of patients in two Médecins Sans Frontières clinics in northern Lebanon	2019	Duplicate
Canada, Citizenship and Immigration: Population Profile : Syrian Refugees	2015	Cannot differentiate populations
Chaudhry: CARING FOR SYRIAN R EFUGEES : A GUIDE FOR PRIMARY CARE PROVIDERS IN ONTARIO – Niagara Region Edition	2015	Wrong article type
Danish Refugee Council: Iraqi Population Survey in Lebanon Iraqi Population Survey in Lebanon	2007	Cannot differentiate populations
Dhital: Dealing with the burden of hypertension in Nepal : current status , challenges and health system issues	2013	Population not directly affected
Dolan: Position Paper - Perspectives on the nutrition response in the Syria crisis	2014	No methods
Doocy: Pilot Testing and Implementation of a mHealth tool for Non-communicable Diseases in a Humanitarian Setting	2017	Cannot differentiate populations

Dookeran: Chronic Disease and Its Risk Factors Among Refugees and Asylees in Massachusetts , 2001-2005	2010	Duplicate
Dudova: Commonest Diseases Amongst Iraqi Internally Displaced After Islamic State Expansion	2015	Duplicate
Farhat: Migrants ' journey , vulnerabilities , access to information and endured violence during the journey and in refugee camps in Ioannina , Attica , Athens and Samos , Greece	2017	Cannot differentiate populations
Ford: Chronic Disease in Health Emergencies : In the Eye of the Hurricane	2006	Population not directly affected
German Foreign Office: Report External Mid-Term Evaluation of Humanitarian Aid. Refugee aid in Lebanon	2017	No methods
Hashani: Lifestyle predictors of hypertension in the adult population of post-war Kosovo	2013	Cannot differentiate populations
Health Sector Cox's Bazar: Rohingya Crisis in Cox ' s Bazar District , Bangladesh : Health Sector Bulletin	2019	No methods
Healthwatch: Health issues of asylum seekers and refugees in Gateshead and Newcastle 2016	2016	Qualitative study talking to general population
Human Rights Watch: VENEZUELA'S HUMANITARIAN CRISIS. Severe Medical and Food Shortages, Inadequate and Repressive Government Response	2016	Qualitative study talking to general population
IPSOS: Healthcare Assessment Of Syrian And Iraqi Urban Refugees In Jordan	2015	No methods
IPSOS: SECOND IPSOS SURVEY ON IRAQI REFUGEES (November 2007)	2007	No methods
John Snow, INC: Health & status of Palestine refugees from Syria in Jordan		Qualitative study talking to general population
Kario: Disaster Hypertension – Its Characteristics, Mechanism, and Management –	2012	Wrong article type
Kayali: Treating Syrian refugees with diabetes and hypertension Frontières model of care and treatment outcomes . Treating Syrian refugees with diabetes and hypertension in Shatila refugee camp , Lebanon : Médecins Sans Frontières model of care and treatment outc	2019	Duplicate
Khader: Cohort monitoring of persons with hypertension : an illustrated example from a primary healthcare clinic for Palestine refugees in Jordan . Cohort monitoring of persons with hypertension : an illustrated example from a primary healthcare clinic for Palest	2019	Duplicate
Khader: Hypertension and treatment outcomes in Palestine refugees in United Nations Relief and Works Agency primary health care clinics in Jordan	2014	Duplicate
Levine: Counting the cost Assessing the cost of ill-health in West Darfur, Sudan	2019	Population not directly affected
Mcnatt: "What's happening in Syria even affects the rocks " : a qualitative study of the Syrian refugee experience accessing	2019	Qualitative study talking

noncommunicable disease services in Jordan		to general population
Médecins Sans Frontières: EAST DARAA, SYRIA BASELINE ASSESSMENT	2016	Cannot differentiate populations
Médecins Sans Frontières: Haiti one year after	2011	No methods
Médecins Sans Frontières: Mixed methods evaluation of MSF primary care based NCD service in Irbid , Jordon : February 2017 - February 2018	2019	Cannot differentiate populations
Médecins Sans Frontières: Morbidity, healthcare needs and barriers to access medical care amongst local and displaced populations in west Dar'aa and Quneitra, Southern Syria	2019	No methods
Médecins Sans Frontières: PROGRAMME EAST DARAA , SYRIA FIRST FOLLOW-UP ASSESSMENT	2017	Cannot differentiate populations
Palafox: Wealth and cardiovascular health : a cross-sectional study of wealth-related inequalities in the awareness , treatment and control of hypertension in high- , middle- and low-income countries	2016	Cannot differentiate populations
Pavli: Health problems of newly arrived migrants and refugees in Europe	2017	Wrong article type
Porte: Evaluation report : Response to Natural Disaster Transversal evaluation of seven OCG interventions in 2007	2008	No methods
Redditt: Health status of newly arrived refugees in Toronto, Ont	2015	Duplicate
Rehr: Prevalence of non-communicable diseases and access to care among non-camp Syrian refugees in northern Jordan	2018	Duplicate
Salazar: Health Consequences of an Armed Conflict in Zamboanga , Philippines Using a Syndromic Surveillance Database	2018	Duplicate
Sethi: Community-Based Noncommunicable Disease Care for Syrian Refugees in Lebanon Global and Regional Refugee Trends	2017	No methods
State of Queensland (Queensland Health): Iraqi Australians	2011	Wrong article type
Su: Refugee Health Needs Assessment in Omaha, Nebraska	2017	Cannot differentiate populations
U.S. Department of Health and Human Services: BHUTANESE REFUGEE HEALTH PROFILE	2014	No methods
U.S. Department of Health and Human Services: SYRIAN REFUGEE HEALTH PROFILE	2016	No methods
United Nations High Commissioner for Refugees: At a glance Health access and utilization survey among non-camp refugees in Jordan	2015	Cannot differentiate populations
United Nations High Commissioner for Refugees: At a glance Health access and utilization survey among Syrian refugees in Lebanon	2016	Cannot differentiate populations
United Nations High Commissioner for Refugees: Directives opérationnelles pour l ' amélioration de la santé des nouveau-nés lors des opérations de secours aux réfugiés Table des matières	2013	Study population selected on the basis of

		another disease
United Nations High Commissioner for Refugees: Forcibly Displaced Myanmar Nationals (FDMNs) in Cox's Bazar , Bangladesh: Health Sector Bulletin	2017	No methods
United Nations High Commissioner for Refugees: HEALTH ACCESS AND UTILIZATION SURVEY - ACCESS TO HEALTH SERVICES IN JORDAN AMONG SYRIAN REFUGEES	2016	Cannot differentiate populations
United Nations High Commissioner for Refugees: Health access and utilization survey among Syrian refugees in Lebanon	2017	Cannot differentiate populations
United Nations High Commissioner for Refugees: Health access and utilization survey among Syrian refugees in Lebanon	2018	Cannot differentiate populations
United Nations High Commissioner for Refugees: Health access and utilization survey. Access to Healthcare Services Among Syrian Refugees in Jordan	2018	Cannot differentiate populations
United Nations High Commissioner for Refugees: Health and Nutrition Quarterly	2014	No methods
United Nations High Commissioner for Refugees: HEALTH CARE IN BOSNIA AND HERZEGOVINA IN THE CONTEXT OF THE RETURN OF REFUGEES AND DISPLACED PERSONS	2001	Wrong article type
United Nations High Commissioner for Refugees: Iraq. Multi-Sector Needs Assessment (MSNA) IV of Refugees Living out of Formal Camps in the KR-I	2019	Cannot differentiate populations
United Nations High Commissioner for Refugees: Joint Assessment for Syrian Refugees in Egypt	2013	Cannot differentiate populations
United Nations High Commissioner for Refugees: Multi-Sector Needs Assessment III (MSNA III) Syrian Refugees in Host Communities of Kurdistan Region of Iraq (KR-I)	2017	Cannot differentiate populations
United Nations High Commissioner for Refugees: MULTI-SECTOR NEEDS ASSESSMENT OF SYRIAN REFUGEES RESIDING IN CAMPS	2015	Cannot differentiate populations
United Nations High Commissioner for Refugees: Operational Upadate - Jordan August 2018	2018	No methods
United Nations High Commissioner for Refugees: RESTORING DIGNITY : RESPONSES TO THE CRITICAL NEEDS OF VULNERABLE PALESTINE REFUGEES	2011	No methods
United Nations High Commissioner for Refugees: Rohingya Crisis in Cox's Bazar District, Bangladesh: Health Sector Bulletin	2019	No methods
United Nations High Commissioner for Refugees: The Annual Report of the Department of Health 2011	2012	No methods
United Nations High Commissioner for Refugees: THE DEMOCRATIC REPUBLIC OF CONGO SITUATION	2018	No methods
United Nations Relief and Works Agency: lebanon field office	2013	No methods
United Nations Relief and Works Agency: UNRWA health department annual report 2012	2013	No methods
United Nations Relief and Works Agency: UNRWA health department annual report 2017	2017	No methods
World Health Organization: Health conditions of , and assistance to , the Arab population in the occupied Arab territories , including	2001	No methods

Palestine		
World Health Organization: Health of refugees and migrants. Regional situation analysis, practices, experiences, lessons learned and ways forward - Africa	2018	Wrong article type
World Health Organization: Health of refugees and migrants. Regional situation analysis, practices, experiences, lessons learned and ways forward -Europe	2018	Wrong article type
World Health Organization: Syrian Arab Republic , Jordan , Lebanon , Iraq	2013	No methods
World Health Organization:Health of refugees and migrants. Practices in addressing the health needs of refugees and migrants	2018	No methods
World Vision Lebanon: Assessment of reduction of water provision in informal tented settlements and its association with the livelihood status of Syrian refugees in the Bekaa Valley.	2018	Cannot differentiate populations
تكلفة الحرب الخفية		Wrong article type
