# Supplementary material for: A systematic review of the burden of hypertension, access to services and patient views of hypertension in humanitarian crisis settings
Source: BMJ Glob Health. 2020 Nov 9;5(11):e002440. doi: 10.1136/bmjgh-2020-002440 (PMC7654140; doi:10.1136/bmjgh-2020-002440)
Supplement: Supplementary data [file bmjgh-2020-002440supp005.pdf]

Appendix 5

Table showing risk of bias across all domains for included studies.

	External validity			Internal validity								
**Author, year**	**A**	**B**	**C**	**D**	**E**	**F**	**G**	**H**	**I**	**Overall external validity ROB**	**Overall internal validity ROB**	**Overall ROB**
**Abukhdeir 2013**(77)	Yes	Unclear	Yes	No	Yes	NA	Yes	Yes	No	Low	High	High
**Adrega 2018**(71)	No	No	NA	Yes	No	No	Yes	Unclear	Yes	High	High	High
**An 2015**(50)	No	No	NA	Yes	No	No	Yes	Yes	Yes	High	High	High
**Anon. 2010**(109)	Yes	Yes	No	Yes	Yes	Unclear	NA	No	Yes	High	High	High
**Balcilar 2016**(79)	Yes	Yes	Yes	Yes	Yes	Yes	Yes	Yes	Yes	Low	Low	Low
**Burger 2019**(61)	No	No	NA	Yes	No	No	Yes	Unclear	Yes	High	High	High
**Burton 2009**(60)	No	NA	NA	No	Yes	No	Yes	Yes	Yes	High	Low	High
**Cetorelli 2017**(68)	Yes	Yes	Yes	No	Yes	No	Yes	Yes	Yes	Low	High	High
**Chahda 2015**(80)	No	Yes	Yes	Yes	No	No	Yes	Unclear	Yes	High	High	High
**Doocy 2013**(63)	No	Yes	Yes	No	No	No	Yes	Unclear	Yes	High	High	High
**Doocy 2015**(82)	Yes	Yes	Yes	No	No	No	Yes	Yes	Yes	Low	High	High
**Doocy 2016 Jordan**(89)	Yes	Yes	Yes	No	Yes	No	Yes	Yes	No	Low	High	High
**Doocy 2016 Lebanon**(83)	No	Yes	Yes	No	Yes	No	Yes	Yes	No	High	High	High
**Doocy 2018**(81)	No	No	No	Yes	No	Unclear	Yes	Yes	Yes	High	High	High
**Dudova 2015**(69)	No	Yes	NA	No	No	No	Yes	Unclear	Yes	High	High	High
**Ebner 2016**(38)	No	Yes	NA	No	No	No	Yes	No	Yes	High	High	High
**Eryurt 2019**(84)	Yes	Yes	NA	Yes	Yes	Yes	Yes	No	No	High	High	High
**Furusawa 2011**(36)	Yes	Yes	NA	Yes	No	No	Yes	No	No	High	High	High
**Gomez 2009**(51)	No	Yes	NA	No	No	No	NA	Unclear	No	High	High	High
**Gomez-Restrepo 2015**(35)	Unclear	No	NA	Yes	Yes	No	Yes	Unclear	Yes	High	High	High
**Greenough 2008**(52)	Yes	Yes	Yes	Yes	No	No	Yes	Yes	Yes	Low	High	High

**Hayashi 2017**(39)	No	Yes	NA	No	No	No	Yes	No	Yes	High	High	High
**Hoshide 2019**(40)	No	No	NA	Yes	Yes	Yes	Yes	Yes	Yes	High	Low	High
**Hurricane Katrina Community Advisory Group 2007**(53)	Yes	Yes	No	Yes	No	No	Yes	Unclear	No	High	High	High
**Islam 2008**(54)	No	NA	No	Yes	Yes	Yes	Yes	Yes	Yes	High	Low	High
**Jen 2015**(31)	No	Yes	Yes	Yes	Yes	No	Yes	Yes	Yes	High	Low	High
**Kayali 2019**(90)	No	Yes	NA	No	Yes	No	Yes	NA	Yes	High	High	High
**Khader 2012**(73)	No	Yes	NA	No	Yes	No	Yes	NA	Yes	High	High	High
**Khader 2014**(72)	No	Yes	NA	No	Yes	No	Yes	NA	Yes	High	High	High
**Krol 2007**(55)	No	Yes	NA	No	No	No	Yes	Unclear	Yes	High	High	High
**Krousel-Wood, 2008**(56)	No	No	NA	Yes	Yes	Yes	Yes	NA	Yes	High	Low	High
**Lafta 2016**(65)	No	Yes	Yes	Yes	No	No	Yes	Yes	Yes	High	High	High
**Lin 2015**(91)	No	No	NA	Yes	No	No	Yes	Unclear	Yes	High	High	High
**Lipsitz 2010**(70)	No	Yes	NA	No	No	No	Yes	Yes	No	High	High	High
**Maldari 2019**(34)	No	No	Yes	Yes	No	No	Yes	Unclear	No	High	High	High
**Mateen 2012**(64)	No	No	NA	No	Yes	No	NA	Unclear	Yes	High	High	High
**Mobula 2016**(78)	No	Yes	NA	No	No	No	yes	yes	No	High	High	High
**Mousa 2010**(74)	No	Yes	NA	Yes	No	No	Yes	No	Yes	High	High	High
**Nagai 2018**(41)	No	Yes	NA	No	No	No	Yes	No	Yes	High	High	High
**Nomura 2016**(42)	No	Yes	NA	No	No	No	Yes	No	Yes	High	High	High
**Ohira 2016**(43)	No	Yes	NA	No	Yes	Yes	Yes	Yes	Yes	High	Low	High
**Prueksaritanond 2007**(62)	No	No	NA	Yes	No	No	NA	Unclear	Yes	High	High	High
**Rehr 2018**(85)	Yes	Yes	Yes	No	No	No	Yes	Yes	Yes	Low	High	High
**Renzaho 2014**(33)	No	No	NA	Yes	Yes	No	Yes	Yes	Yes	High	Low	High
**Rodriguez 2006**(57)	No	No	NA	Yes	No	No	Yes	Unclear	No	High	High	High
**Saadeh 2015**(75)	No	yes	NA	No	No	No	Yes	Yes	Yes	High	High	High
**Sakai 2017**(44)	No	Yes	NA	No	No	No	Yes	Yes	No	High	High	High
**Saleh 2018**(76)	No	Yes	Yes	Yes	No	No	Yes	No	Yes	High	High	High
**Satoh 2016**(45)	No	Yes	NA	Yes	Yes	No	Yes	Yes	No	High	High	High
**Shiba 2019**(46)	Yes	No	No	Yes	Yes	No	Yes	Yes	Yes	High	High	High
**Suda 2019**(47)	No	Yes	NA	No	Yes	Unclear	Yes	Unclear	No	High	High	High
**Sun 2013**(92)	Yes	Yes	NA	Yes	No	No	Yes	Yes	Yes	High	High	High
**Takahashi 2016**(48)	No	Yes	NA	Yes	No	No	Yes	Unc	Yes	High	High	High

								lear				
**Taylor 2014**(32)	No	Yes	No	Yes	No	No	Yes	Unclear	No	High	High	High
**Toda 2017**(49)	No	Yes	NA	Yes	Yes	Yes	Yes	Yes	Yes	High	Low	High
**UNHCR 2014**(87)	Yes	No	Yes	Unclear	No	No	Yes	Unclear	Yes	High	High	High
**Vanasse 2016**(37)	Yes	Yes	NA	No	No	No	Yes	Yes	Yes	Low	High	High
**Vernier 2019**(88)	Yes	Yes	Yes	No	No	No	Yes	Yes	Yes	Low	High	High
**Vest 2006**(58)	No	Yes	No	Yes	No	No	Yes	Unclear	Yes	High	High	High

A= Was the sampling frame a true or close representation of the target population?
B= Was some form of random selection used to select the sample, or was a census undertaken?
C= Was the likelihood of non-response bias minimal?
D= Were data collected directly from the subjects (as opposed to a proxy)?
E= Was an acceptable case definition used?
F= Was the study instrument that measured the parameter of interest shown to have reliability and validity?
G= Was the same mode of data collection used for all subjects?
H= Was the length of the shortest prevalence period for the parameter of interest appropriate?
I= Were the numerator and denominator for the parameter of interest appropriate?
